# Empowering Flexible Electronics with Piezoelectric Nanogenerators: Breakthroughs from Energy Harvesting to Intelligent Sensing

**DOI:** 10.1002/advs.202519604

**Published:** 2025-12-27

**Authors:** Wu‐Lin Xin, He‐Qing Cai, Xi Cui, Lu Han, Kou Zhang, Xin‐Yu Xue, Yi‐Fei Song, Juan Liu, Zhou Li

**Affiliations:** ^1^ Beijing Engineering Research Center of Printed Electronics Beijing Institute of Graphic Communication Beijing 102600 China; ^2^ Hepato‐Pancreato‐Biliary Center Beijing Tsinghua Changgung Hospital School of Clinical Medicine Tsinghua Medicine Tsinghua University Beijing 102218 China; ^3^ Vita Tech Innovation Center Tsinghua Changgung Hospital School of Clinical Medicine Tsinghua University Beijing 100084 China; ^4^ School of Biomedical Engineering Tsinghua University Beijing 100084 China

**Keywords:** energy harvesting, flexible sensor, piezoelectric nanogenerator, self‐charging supercapacitor

## Abstract

Flexible sensing devices and energy storage systems with self‐powered capabilities are propelling the rapid advancement of flexible electronics and wearable technologies. Piezoelectric nanogenerators (PENGs) present a compelling alternative to the constraints of conventional battery‐powered systems, which suffer from limited capacity and short lifespans. By exploiting the piezoelectric effect, PENGs convert mechanical energy into electrical energy without the need for an external power source, producing electricity in response to mechanical stimuli, including vibration, pressure, and force. When integrated into flexible electronics and sensors, PENGs facilitate applications such as health monitoring, bionic electronic skin, and tactile sensing. Furthermore, PENGs can be combined with energy storage systems such as self‐recharging supercapacitors and batteries, enhancing energy harvesting and conversion, while promoting sustainable energy utilization and ensuring a reliable power supply. This paper reviews recent advancements in PENGs, highlighting their theoretical foundation, structural design, and potential applications in sensors and energy storage systems, and discusses potential future directions for their continued advancement.

## Graphical abstract

This review systematically summarizes recent advances in self‐powered flexible sensing and energy storage systems based on piezoelectric nanogenerators (PENGs). It highlights their fundamental mechanisms, structural designs, and applications in health monitoring, e‐skin, and tactile sensing. The integration of PENGs with supercapacitors or batteries is also discussed for enabling sustainable and autonomous wearable electronics.

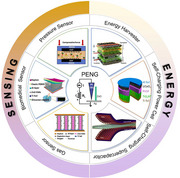

## 1 Introduction

Advancements in contemporary technology and societal progress have ushered in an era characterized by intelligence and connectivity. The Internet of Things (IoT)^[^ ^1^ ^]^ has been extensively integrated into smart wearables,^[^ ^2^ ^]^ healthcare systems,^[^ ^3^ ^]^ smart homes,^[^ ^4^ ^]^ and environmental monitoring,^[^ ^5^ ^]^ collectively enhancing quality of life. Nevertheless, despite their widespread adoption, traditional battery‐powered solutions face persistent challenges, particularly in terms of capacity, lifespan, and the long‐term sustainability of energy supply.^[^ ^6^ ^]^

A promising approach to overcome the energy‐supply and performance limitations of flexible electronics is the PENG device. These low‐power, self‐powered devices generate electrical output through mechanical deformation induced by external forces,^[^ ^7^, ^8^ ^]^ converting mechanical energy—such as vibrations, pressure, or human motion^[^ ^9^ ^]^—into electrical energy,^[^ ^10^ ^]^ while simultaneously enabling self‐powered sensing.^[^ ^11^ ^]^

**Figure 1:**
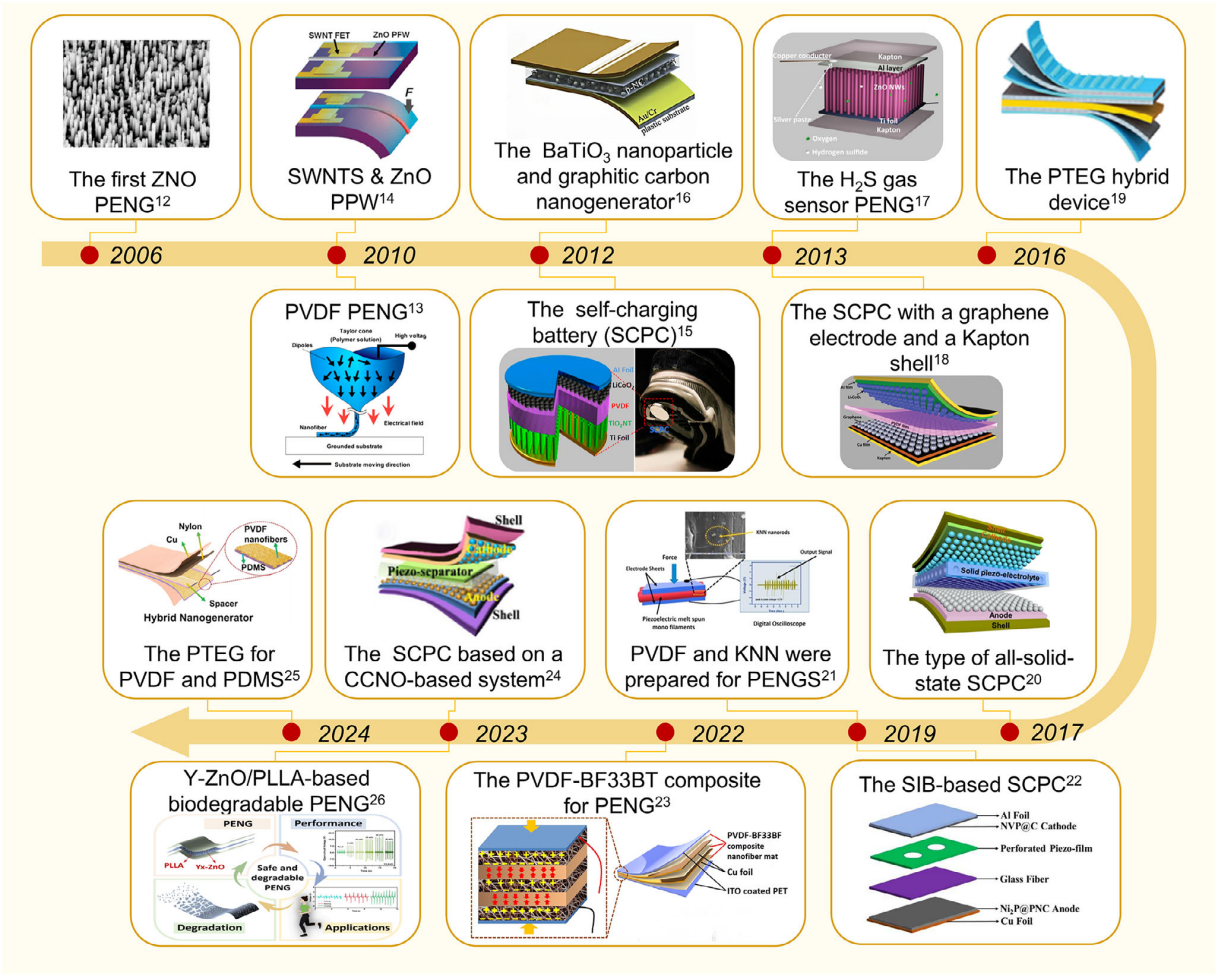
Timeline of advancements in piezoelectric nanogenerators (PENGs) and self‐charging systems (SCPC). Reproduced with permission.^[^ ^12^ ^]^ Copyright 2006, AAAS. Reproduced with permission.^[^ ^13^ ^]^ Copyright 2010, American Chemical Society. Reproduced with permission.^[^ ^14^ ^]^ Copyright 2010, American Chemical Society. Reproduced with permission.^[^ ^15^ ^]^ Copyright 2012, American Chemical Society. Reproduced with permission.^[^ ^16^ ^]^ Copyright 2012, Wiley–VCH. Reproduced with permission.^[^ ^17^ ^]^ Copyright 2013, IOP Publishing. Reproduced with permission.^[^ ^18^ ^]^ Copyright 2013, Wiley–VCH. Reproduced with permission.^[^ ^19^ ^]^ Copyright 2016, Elsevier. Reproduced with permission.^[^ ^20^ ^]^ Copyright 2017, Elsevier. Reproduced with permission.^[^ ^21^ ^]^ Copyright 2019, Elsevier. Reproduced with permission.^[^ ^22^ ^]^ Copyright 2019, Elsevier. Reproduced with permission.^[^ ^23^ ^]^ Copyright 2022, American Chemical Society. Reproduced with permission.^[^ ^24^ ^]^ Copyright 2023, American Chemical Society. Reproduced with permission.^[^ ^25^ ^]^ Copyright 2024, American Chemical Society. Reproduced with permission.^[^ ^26^ ^]^ Copyright 2024, American Chemical Society.

Since their seminal demonstration in 2006, research on PENGs has evolved substantially in both methodology and conceptual frameworks, progressing from foundational investigations into piezoelectric phenomena to multifunctional systems with cross‐domain interoperability, as shown chronologically in **Figure** **1**. Early implementations predominantly employed vertically aligned zinc oxide (ZnO) nanowire configurations, laying the groundwork for proof‐of‐concept demonstrations of microscale energy transduction via piezoelectric coupling under mechanical excitation. Subsequent research has refined these systems through continuous advancements in piezoelectric material engineering and device‐architecture optimization.

Notably, in 2013, a flexible multilayer stacking structure was proposed, replacing rigid device components with Kapton shells and graphene electrodes, which significantly enhanced output performance. Since 2017, PENG research has increasingly focused on integrating novel composite materials and multifunctional designs. A significant breakthrough occurred in 2019 with the proposal to enhance sodium‐ion batteries (SIBs) by incorporating perforated piezoelectric materials into elastic SIB structures, substantially improving both energy density and cycling stability. Between 2021 and 2025, further advancements have continued to improve the power‐generation capabilities and operational stability of PENGs. These developments have enabled PENGs to achieve greater environmental adaptability and higher energy‐conversion efficiency, thereby laying a solid foundation for their deployment across diverse application scenarios.

Furthermore, the selection of high‐performance piezoelectric materials has remained pivotal in advancing energy harvesting capabilities. For instance, nanoscale materials, such as ZnO nanowires^[^ ^27^, ^28^ ^]^ and polyvinylidene fluoride (PVDF),^[^ ^28^, ^29^, ^30^ ^]^ frequently exhibit superior performance compared to their macroscopic counterparts. This is primarily due to the ability of nanoscale piezoelectric materials to generate substantial current outputs even with limited dimensions and minor deformations, thereby facilitating more efficient energy harvesting. PENG's flexible nanogenerators utilize these piezoelectric materials, enabling a broad spectrum of applications owing to their remarkable flexibility, reliability, processability, and cost‐effectiveness.^[^ ^31^, ^32^, ^33^, ^34^, ^35^ ^]^ For example, they can act as continuous power sources in piezoelectric sensing applications, delivering power to smart bracelets and detection systems.^[^ ^36^ ^]^ This self‐driven energy source not only reduces reliance on conventional batteries but also enhances both the functionality and environmental sustainability of such devices. Additionally, these materials can be integrated into garments or fabricated to create electronic skin for biosensing applications,^[^ ^37^ ^]^ thereby offering a promising avenue for telemedicine applications.^[^ ^38^, ^39^, ^40^ ^]^ They can also be employed in gas sensing to assess air quality by detecting key indicators within indoor environments, such as carbon dioxide levels, air humidity, and other environmental factors.^[^ ^41^ ^]^

Despite notable advancements in the theoretical and experimental research of flexible sensors and energy storage systems based on PENGs, challenges persist in ensuring long‐term energy recyclability, material durability, and environmental sustainability in practical applications.^[^ ^42^, ^43^, ^44^ ^]^ A pivotal advancement involves integrating PENGs with energy storage devices, such as energy harvesters, self‐charging supercapacitors, and self‐rechargeable batteries. PENGs function not only as “energy capturers” but also as “energy sustainers.” By integrating PENGs with microbial fuel cells, which harness environmental vibrations to maintain battery charge, these systems can operate sustainably without the need for an external power source.^[^ ^45^, ^46^ ^]^ This allows self‐rechargeable batteries to significantly enhance the sustainability, capacity, and rapid charging capabilities of the energy system, providing a long‐lasting and reliable energy supply. By combining PENGs with supercapacitors, energy generated from human motion can be efficiently replenished. Self‐charging batteries and self‐charging supercapacitors are at the forefront of energy storage technology, delivering significant improvements in energy harvesting and storage efficiency. The harvested energy is utilized across diverse applications during the processes of energy exchange and storage.^[^ ^47^ ^]^ When paired with PENGs, self‐charging supercapacitors—known for their high power density and rapid charging and discharging capabilities^[^ ^48^, ^49^ ^]^—enhance instantaneous energy storage efficiency by promptly transforming ambient mechanical energy into usable electrical energy. The integration of PENGs with self‐charging supercapacitors is particularly effective for harvesting human motion energy, rendering them particularly suitable for applications in wearable technology.^[^ ^30^, ^50^ ^]^ This enables greater design flexibility for low‐power devices and provides sustained energy support for smart devices and health monitoring systems.

**Figure 2:**
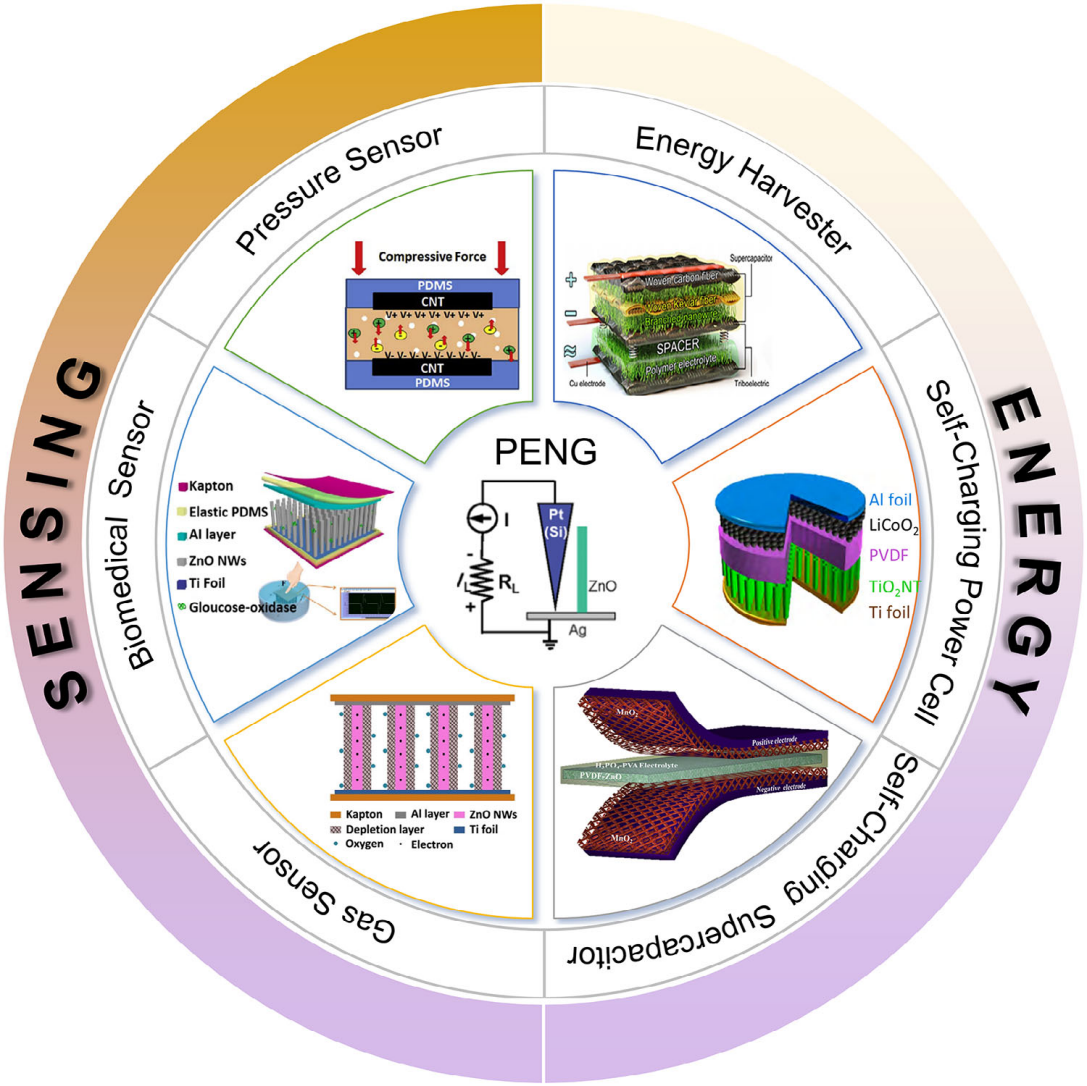
Overview of multifunctional self‐supply system based on PENG technology. Reproduced with permission.^[^ ^12^ ^]^ Copyright 2006, AAAS. Adapted with permission.^[^ ^51^ ^]^ Copyright 2016, Elsevier. Adapted with permission.^[^ ^52^ ^]^ Copyright 2016, Elsevier. Adapted with permission.^[^ ^17^ ^]^ Copyright 2012, IOP Publishing. Adapted with permission.^[^ ^53^ ^]^ Copyright 2020, Elsevier. Adapted with permission.^[^ ^15^ ^]^ Copyright 2012, American Chemical Society. Adapted with permission.^[^ ^54^ ^]^ Copyright 2015, American Chemical Society.

This review examines the selection and optimization of piezoelectric materials, structural designs, and system‐integration technologies, and provides a comprehensive overview of recent advances in PENGs for flexible self‐powered sensing, environmental monitoring, and advanced energy‐storage systems. Specifically, it covers the fundamental operating principles of PENGs, multimodal hybrid energy harvesting, flexible sensor architectures, and integrated energy systems incorporating self‐charging power units (SCPCs/SCSPCs), thereby elucidating the developmental pathway from materials and devices to system‐level applications. To visually summarize the research framework, a schematic overview is provided (**Figure** **2**) that highlights the core components, flexible sensor applications, energy‐harvesting and storage integration, and system‐level self‐powered platforms. Additionally, we analyze key challenges related to material performance, output stability, environmental adaptability, and system integration, and we propose interdisciplinary research directions, including multimodal energy fusion, structural optimization, and strategies for integrating energy storage. Overall, this review synthesizes the current state of PENG applications in flexible sensing and self‐powered platforms, and provides a systematic, forward‐looking reference for future research and practical implementation.

## 2 Working Mechanism of PENG

In 1880, Pierre Curie and Jacques Curie made the seminal discovery of the piezoelectric effect during their investigation of crystals like quartz, tourmaline, and Rochelle salt.^[^ ^55^ ^]^ The piezoelectric effect is categorized into two distinct phenomena: the direct effect and the converse effect. The converse piezoelectric effect, in which the application of an electric potential leads to mechanical displacement within the material, is relevant to applications such as actuators, acoustic emitters, and vibration damping, and will not be discussed herein.^[^ ^56^ ^]^ The primary focus of this paper is the direct piezoelectric effect, which forms the fundamental basis for the operation of PENGs.^[^ ^57^ ^]^ This effect involves the polarization of materials subjected to tensile or compressive stress.

**Figure 3:**
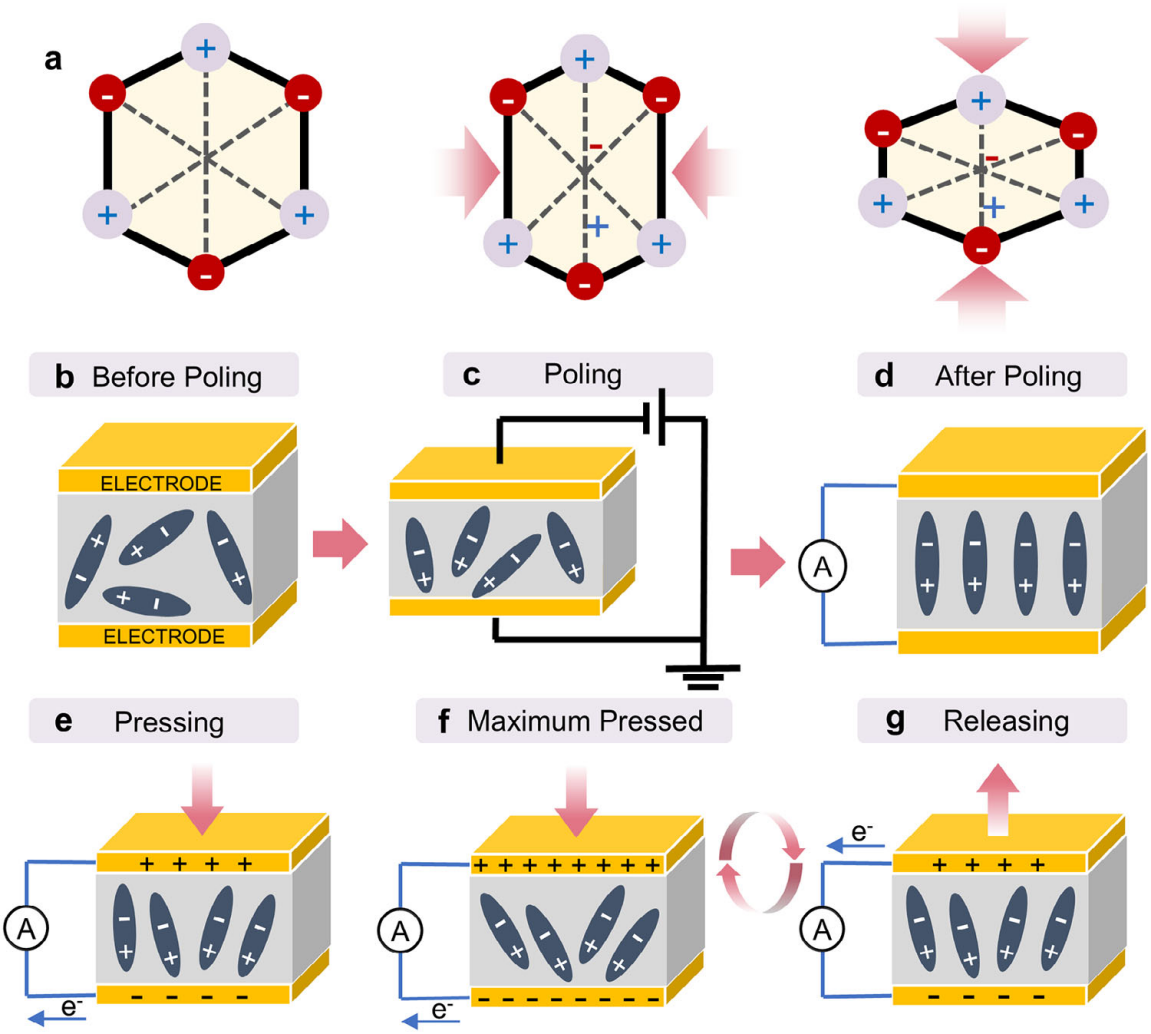
Working mechanism of PENG. a) The piezoelectric effect, taking piezoelectric crystals as an example. b) The state of dipoles before the poling process. c) Poling of a piezoelectric material. d) The dipole makes placed alignment in a regular direction after the poling process. e,f) The piezopotential occurred after the application of compressive forces and reached the maximum polarization density. g) Electronics flow back to equilibrium after the force is released.

As depicted in **Figure** **3**a, a non‐centrosymmetric molecule, whose centers of negative and positive charges coincide in a neutral state, develops a dipole upon the application of stress, resulting in the separation of these charge centers. This phenomenon illustrates the mechanism by which pressure or deformation generates an electric field within piezoelectric materials.^[^ ^32^ ^]^ Among the 32 crystallographic classes, 21 are non‐centrosymmetric, lacking a center of symmetry, and 20 of these exhibit direct piezoelectricity, with the cubic class being the exception.^[^ ^8^ ^]^ In these non‐centrosymmetric materials, the absence of symmetry in ion distribution results in the formation of electrical dipoles, producing a piezoelectric response. As illustrated in Figure 3b, in most other materials, molecular dipoles are randomly oriented within their crystal structure, thereby nullifying any appreciable piezoelectric effect. To induce an effective piezoelectric response in such materials, a process termed “poling” is employed.^[^ ^58^ ^]^ As shown in Figure 3c, this process involves reorienting molecular dipoles by applying a high electric field under elevated temperatures, followed by cooling while maintaining the electric field to preserve the alignment of the dipoles.^[^ ^59^, ^60^ ^]^

Upon the application of mechanical stress (Δσ), the charge (Q) produced on the opposing surfaces of a piezoelectric material with an area (A) is defined by Equation (1)^[^ ^61^ ^]^:

(1) $$Q=d_{33}\cdot A\cdot \Delta \sigma$$

where (*d*_33_) is the material's piezoelectric coefficient in the 33‐mode. Under open circuit conditions, where the load impedance is considered infinite, the relationship (Q = C · V) applies, where (C $=A\cdot \frac{\varepsilon_{33}^{T}}{h}$) is the material capacitance. This allows the voltage (V) to be determined from Equation (2):

(2) $$V=\frac{d_{33}}{\varepsilon_{33}^{T}}\cdot h\cdot \Delta \sigma$$

where (h) represents the thickness and ($\varepsilon_{33}^{T}$) denotes the permittivity at constant stress in the polarization direction. The energy (E) stored in a capacitor, resulting from the applied stress, is expressed by Equation (3):

(3) $$E=\frac{1}{2}\cdot \frac{{d_{33}}^{2}}{\varepsilon_{33}^{T}}\cdot A\cdot h\cdot \;{\left(\Delta \sigma \right)}^{2}$$

Thus, for a given thickness and area, the theoretical maximum output energy scales linearly with ($\frac{{d_{33}}^{2}}{\varepsilon_{33}^{T}}$), which is known as the harvesting figure of merit for evaluating a material's performance in off‐resonance vibration harvesting. Under short circuit conditions, the current (I) is defined by ($I\;=\frac{dQ}{dt}$) and can be written as Equation (4):

(4) $$I=d_{33}\cdot A\cdot \frac{d\left(\Delta \sigma \right)}{dt}$$

The coupling coefficient (k), which represents the efficiency of energy conversion in generator mode, is defined by Equation (5)^[^ ^62^ ^]^:

(5) $$k^{2}=\frac{\mathrm{transformed}\;\mathrm{energy}}{\mathrm{incoming}\;\mathrm{energy}}$$

The coupling coefficients in the longitudinal mode ( *k*_33_) and transversal mode (*k*_31_) are defined as follows:

(6) $$\;k_{33}=\frac{d_{33}}{\sqrt{\varepsilon_{33}^{T}\cdot s_{33}^{E}}}$$

(7) $$k_{31}=\frac{d_{31}}{\sqrt{\varepsilon_{33}^{T}\cdot s_{11}^{E}}}$$

A higher coupling coefficient (k) reflects an enhanced ability to harvest mechanical energy. **Table** **1** summarizes the properties of frequently utilized piezoelectric materials.

**Table 1:** Properties of part piezoelectric materials.

Piezoelectric material	Relative permittivity [ɛ_r_]	Piezoelectric constant, d_33_ [pC N^−1^]	Electromechanical coupling factor [k_33_]
PVDF	6 to 12^[^ ^64^ ^]^	−24 to −34^[^ ^65^ ^]^	0.20^[^ ^66^ ^]^
P (VDF‐co‐TrFE)	18^[^ ^67^ ^]^	−25^[^ ^68^ ^]^	0.29^[^ ^66^ ^]^
P(VDF‐co‐HFP)	11^[^ ^69^ ^]^	−24^[^ ^70^ ^]^	0.36^[^ ^69^ ^]^
P(VDF‐co‐CTFE)	13^[^ ^71^ ^]^	−140^[^ ^72^ ^]^	0.36^[^ ^73^ ^]^
ZnO	10.9^[^ ^74^ ^]^	12.4^[^ ^74^, ^75^ ^]^	0.48^[^ ^74^ ^]^
BaTiO_3_	1200^[^ ^76^ ^]^	149^[^ ^76^ ^]^	0.49^[^ ^77^ ^]^
LiNbO_3_	28.7^[^ ^78^ ^]^	6^[^ ^78^ ^]^	0.23^[^ ^79^ ^]^
PZT	1300^[^ ^80^ ^]^	225 to 590^[^ ^80^ ^]^	0.7^[^ ^81^ ^]^
KNN	496^[^ ^80^ ^]^	80 to 160^[^ ^82^ ^]^	0.51^[^ ^83^ ^]^
BNT	356^[^ ^84^ ^]^	89^[^ ^84^ ^]^	≈0.21^[^ ^84^ ^]^

Traditional piezoelectric materials can be categorized into piezoelectric crystals, ceramics, polymers, and composites. Ferroelectric oxides, such as Barium Titanate (BTO) and Lead Zirconate Titanate (PZT), exhibit high piezoelectric coefficients and electromechanical coupling but demonstrate limited mechanical flexibility. In contrast, piezoelectric polymers are highly flexible but have low piezoelectric coefficients. Piezoelectric nanocomposites, which incorporate inorganic fillers within a polymer matrix, emerge as promising candidates for PENGs, combining polymer flexibility with the strong piezoelectric performance of inorganic fillers.^[^ ^63^ ^]^

The concept of PENG was initially demonstrated in 2006, showcasing the conversion of mechanical energy into electricity via the deflection of ZnO nanowires using a conductive atomic force microscopy (AFM) tip in contact mode.^[^ ^12^ ^]^ Typically, PENGs consist of piezoelectric materials and electrodes, where the application of stress or deformation on the piezoelectric material surface induces charge generation. This charge is transmitted as current through an external load upon connection of the PENG.^[^ ^85^ ^]^ Figure 3d–g provides a schematic representation of the operational mechanism of PENGs. Initially, when the PENG is in its natural state, no measurable electrical signal is generated (Figure 3d). Upon the application of vertical pressure, negative strain induces electric dipoles due to the displacement of charge centers of cations and anions, resulting in the formation of equivalent positive and negative piezoelectric potentials at the extremities of the material (Figure 3e). This induces a current as electrons migrate between electrodes to neutralize the electric field until potential balance is achieved (Figure 3f). Upon the removal of pressure, the PENG recovers from its bent state to its original state, causing a reduction in piezoelectric potential and a reversal in electron flow until full recovery (Figure 3g).^[^ ^86^ ^]^

In the field of flexible electronics, PENGs, TENGs, and electromagnetic generators (EMGs) exhibit distinct performance advantages owing to their distinct energy‐conversion mechanisms. PENGs possess ultralow internal resistance and a stable output voltage; their signal profiles are highly predictable, making them suitable for pressure sensors and micropower devices. Huang et al. reported a cellulose/BaTiO_3_ aerogel‐based PENG that delivered 15.5 V and 11.8 µW; when coupled with a TENG, the output power increased to 85 µW.^[^ ^87^ ^]^

In contrast, TENGs rely on contact electrification and deliver high output power at low frequencies (≤ 4 Hz) in contexts such as human motion and environmental vibrations.^[^ ^88^ ^]^ They have been extensively employed for wearable kineticenergy harvesting and self‐powered interactive interfaces. However, their output typically features high‐voltage pulses with large current fluctuations, thereby requiring complex power‐management circuits. Furthermore, the durability of frictional interfaces remains a critical challenge, limiting the reliability of TENGs in flexible systems.^[^ ^89^ ^]^

EMGs exhibit superior power density under macroscopic, high‐frequency vibrations, making them suitable for mechanicalvibration energy harvesting and structural monitoring. However, their dependence on magnets and coils hinders the realization of lightweight, flexible architectures, thereby limiting their applicability in wearable systems.^[^ ^90^ ^]^ Although hybrid energy‐harvesting mechanisms that combine piezoelectric, triboelectric, and electromagnetic conversions can broaden the operating bandwidth and enhance overall power output, system complexity and fabrication challenges remain major bottlenecks.

In summary, PENGs offer the most balanced set of advantages for flexible and wearable self‐powered sensing, owing to their stable outputs, mechanical compliance, and ease of encapsulation and integration. TENGs are more appropriate for low‐frequency energy harvesting and tactile human–machine interaction, whereas EMGs retain their strengths in recovering energy from structural vibrations. These distinctions underscore the irreplaceable role of PENGs in intelligent, flexible sensing systems and offer important guidance for the design of future multimodal, synergistic energy‐harvesting systems.

## 3 Flexible Sensors Based on PENG

The rapid development of flexible electronics, wearable devices, and the IoT has created an urgent demand for self‐powered, high‐sensitivity sensors. PENGs, which integrate energy harvesting and signal transduction, constitute a key solution to this need. This chapter provides a systematic review of recent advances, technical innovations, and practical applications in PENG‐based flexible sensing systems. The chapter first addresses the foundational elements of PENG sensors—including core materials and hybrid energy‐harvesting mechanisms that enable self‐powering—thereby establishing the basis for high‐performance sensing. Next, we explore diverse applications: structural designs that enhance sensitivity in pressure and biomechanical monitoring; skin‐interfaced biosensors and metabolite detection in biomedical diagnostics; and strategies for optimizing selectivity and stability in environmental and hazardousgas sensing. Finally, we discuss the integration of PENGs into intelligent systems, in which energy management, multimodal sensing fusion, and AIenabled signal processing support autonomous operation. By synthesizing the evolution of PENG sensors from fundamental principles to application‐focused designs, this chapter offers a comprehensive reference for researchers in flexible electronics and self‐powered sensing.

### 3.1 Fundamental Components and Working Principles

#### 3.1.1 Core Elements of Piezoelectric Sensors

Piezoelectric sensors primarily consist of flexible materials, conductive elements, and compliant substrates. These components play a pivotal role in facilitating data transmission, information acquisition, and signal transduction.^[^ ^91^ ^]^ Owing to their inherent flexibility, piezoelectric sensors can be seamlessly integrated into various wearable technologies and electronic skin applications, demonstrating their potential for a wide range of applications. Despite the advantages of traditional piezoelectric sensors, such as high sensitivity and robust stability, their progress in miniaturization, intelligence, and integration is frequently limited by intrinsic constraints, including material durability and device responsiveness.^[^ ^92^, ^93^ ^]^

#### 3.1.2 Hybrid Energy Harvesting Mechanisms

To address these challenges, piezoelectric sensors based on PENGs have been developed by integrating the principles of electrostatic induction and the piezoelectric effect for signal monitoring.^[^ ^94^ ^]^ Furthermore, self‐powered capacitors are frequently utilized in piezoelectric sensor applications to minimize dependence on external power sources, thus improving device autonomy. For instance, Evgenia et al.^[^ ^95^ ^]^ developed a self‐powered capacitor for piezoelectric‐driven sensing applications, which employs a poly(vinylidene fluoride‐trifluoroethylene) (PVDF‐TrFE) piezoelectric thin film to harvest and store electrical energy. This capacitor demonstrated the ability to charge up to 0.5 volts within 20 s and achieved a peak voltage of 0.65 volts, making it highly suitable for wearable electronics and IoT sensors.

**Figure 4:**
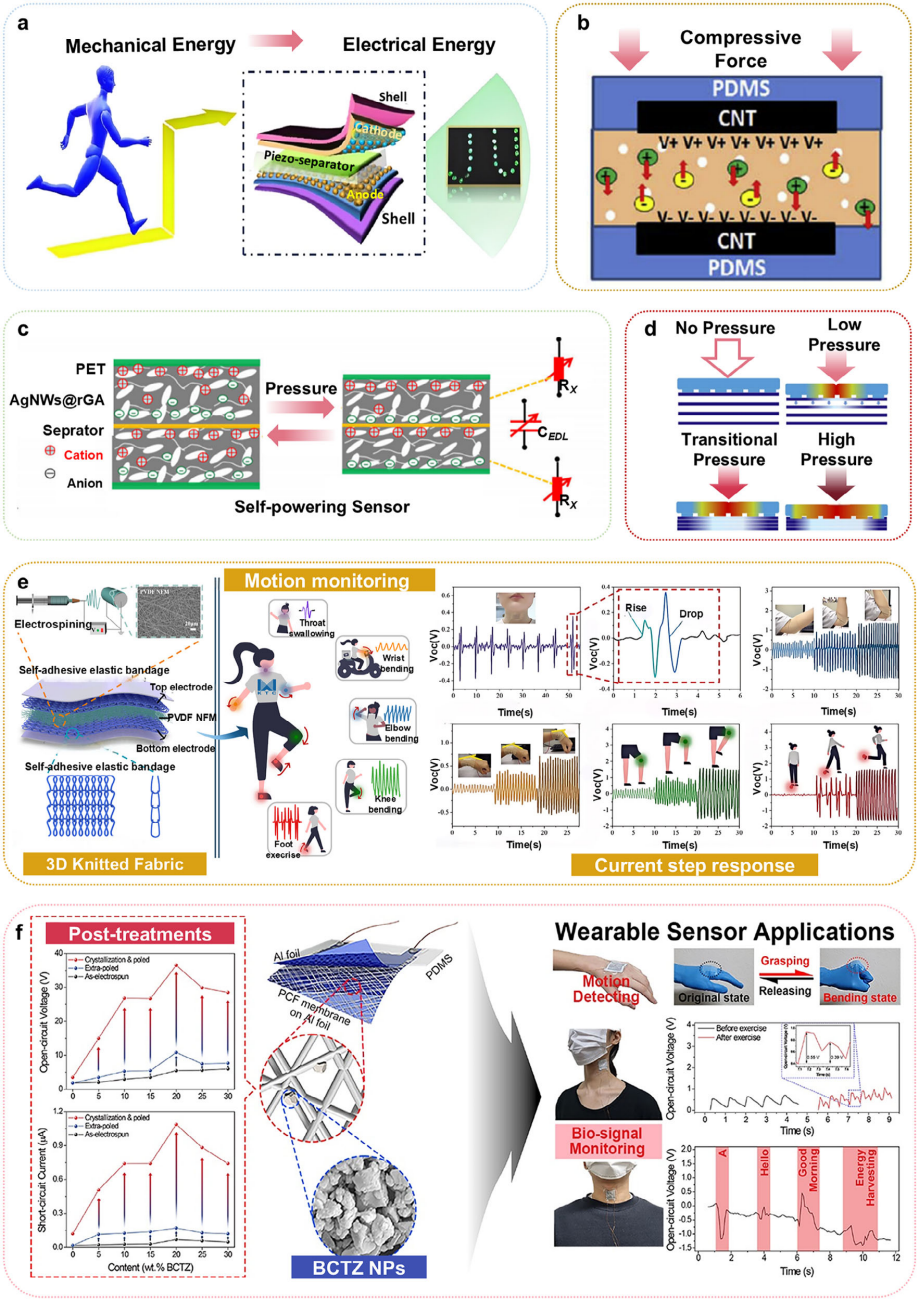
a) A piezoelectric self‐charging flexible supercapacitor (PSCFS) device schematic diagram. Adapted with permission.^[^ ^24^ ^]^ Copyright 2023, American Chemical Society. b) When mechanical force is applied, an electric field is produced that allows ions to migrate according to their characteristic charge. Adapted with permission.^[^ ^51^ ^]^ Copyright 2016, Elsevier. c) The piezoionic self‐powering sensor's operational mechanism. Adapted under the terms of the CC‐BY 4.0 license.^[^ ^96^ ^]^ Copyright 2022, The Authors. d) Diagrammatic representation of the cylindrical microstructure's sensing mechanism at various pressure stages. Adapted with permission.^[^ ^101^ ^]^ Copyright 2023, IOP Publishing. e) ATPS in motion monitoring and intelligent interaction: its structure, preparation method, and application. Adapted with permission.^[^ ^99^ ^]^ Copyright 2024, American Chemical Society. f) Improving the piezoelectric properties of lead‐free BCTZ/PVDF‐TrFE composite fiber membranes and using ultrathin flexible energy harvesters in biomachine energy harvesting. Adapted with permission.^[^ ^100^ ^]^ Copyright 2022, American Chemical Society.

Another innovative approach was developed by Mondal et al.,^[^ ^24^ ^]^ who proposed a piezoelectric‐driven self‐charging supercapacitor based on copper–cobalt–nickel oxide (CCNO), as illustrated in **Figure** **4**a. This system employs a composite material, fabricated from a PVDF polymer matrix integrated with CCNO nanomaterials (PNCU). The PNCU‐1 thin film serves a dual purpose as both the piezoelectric material and the separator, with the electrolyte gel comprising polyvinyl alcohol and phosphoric acid. The positive and negative electrodes are fabricated by depositing CCNO nanowires onto carbon cloth substrates. This advanced structural design allows the storage device to autonomously store electrical energy during mechanical deformations, such as bending and stretching. Notably, after undergoing 10,000 cycles of operation, the system demonstrated exceptional stability, maintaining 98% of its initial capacitance, with only a 9% decrease in performance.

Recent research has shifted from single‐material piezoelectrics to multimaterial systems and composite architectures. The most promising technical strategies center on the development of high‐performance nanostructured piezoelectric materials, the optimization of micro‐ and nanoscale architectures and flexible substrates, and the integration of complementary energy‐harvesting mechanisms, including triboelectric and electrostatic effects. These strategies enable efficient, flexible, and environmentally robust self‐powered sensors, thereby underpinning applications in pressure sensing, biomechanical monitoring, and environmental sensing.

### 3.2 Pressure Sensing and Biomechanical Monitoring

#### 3.2.1 Self‐Powered Pressure Detection Systems

Flexible supercapacitors driven by piezoelectric films offer an alternative approach to energy storage via electrochemical charging. These devices dynamically respond to variations in piezoelectric output signals, rendering them suitable for real‐time stress monitoring in pressure sensors. Furthermore, PENGs demonstrate high strain sensitivity, allowing their output response to simultaneously facilitate stress detection and energy harvesting. As depicted in Figure 4b, a rapid self‐powered, self‐charging electrochemical energy storage device employs carbon nanotube (CNT) electrodes to facilitate ion adsorption and desorption, enabling energy storage from minor mechanical perturbations within seconds.^[^ ^51^ ^]^ The mechanism of action, as shown in Figure 4c, features a novel pressure‐ion self‐powered sensor incorporating highly elastic silver nanowires combined with a reduced graphene aerogel composite.^[^ ^96^ ^]^ This multifunctional device serves as both a pressure sensor and an energy‐storing compressible device. When external pressure deforms the internal structure, the electron transport channel is elongated, causing the reorganization of positive and negative ions and the renewal of the electrical double layer. This mechanism facilitates efficient energy storage and signal transduction.

Moreover, a cylindrical high‐performance flexible piezoresistive pressure sensor with tactile and graphical sensing capabilities has been fabricated, as shown in Figure 4d. This sensor monitors resistance changes under varying pressure conditions, exhibiting high sensitivity to diverse human activities.^[^ ^97^ ^]^ As the pressure increases from the absence of external force to the high‐pressure range (20–80 kPa), the MXene film experiences significant deformation, reducing interatomic spacing and shrinking the gaps between layers. This reduction enhances the sensor's overall performance by decreasing electrical resistance and amplifying the output current.

#### 3.2.2 Wearable Motion and Physiological Sensors

In wearable sensing technologies, Iqra et al.^[^ ^98^ ^]^ introduced an innovative insole‐type piezoelectric nanogenerator fabricated using graphene nanopowder, piezoelectric nanoparticles (BaTiO_3_, ZnO, and PZT), and cost‐effective multi‐walled carbon nanotube (MWCNT)/silicone rubber composite electrodes. This device successfully generated a voltage of 27 V and a current of 0.429 mA during normal walking or running, offering the possibility for a variety of sensor applications.

Figure 4e depicts a 3D, self‐adhesive, all‐textile piezoelectric sensor (ATPS) manufactured using electrospinning and flexible knitting techniques.^[^ ^99^ ^]^ It comprises three functional layers: a stretchable, conductive, 3D knitted fabric on the top, an elastic and adhesive bandage at the bottom, and a PVDF nanofiber membrane (PVDF NFM) electrospun directly onto the knitted substrate in the middle. This configuration offers excellent skin compatibility, mechanical flexibility, and breathability, facilitating reliable and precise monitoring of diverse physiological signals such as swallowing, joint movement, and locomotion. Furthermore, under mechanical deformation, the PVDF nanofiber layer generates piezoelectric signals, enabling autonomous sensing without reliance on external power sources.

Figure 4f depicts a wearable piezoelectric energy harvester incorporating lead‐free BCTZ ceramic nanoparticles and poly(vinylidene fluoride‐trifluoroethylene) [P(VDF‐TrFE)] composite fibers.^[^ ^100^ ^]^ The study demonstrates that post‐processing techniques, such as thermally induced crystallization and polarization treatment, substantially improve β‐phase crystallinity and dipole alignment at the molecular scale, thereby enhancing piezoelectric output performance. Moreover, the system exhibits remarkable flexibility, biocompatibility, and mechanical adaptability, enabling effective detection and self‐powered sensing of various biological signals, including gripping, vocalization, and knee bending. These results highlight its broad application potential in self‐powered wearable sensing and physiological signal monitoring.

#### 3.2.3 Structural Innovations for Enhanced Sensitivity

**Figure 5:**
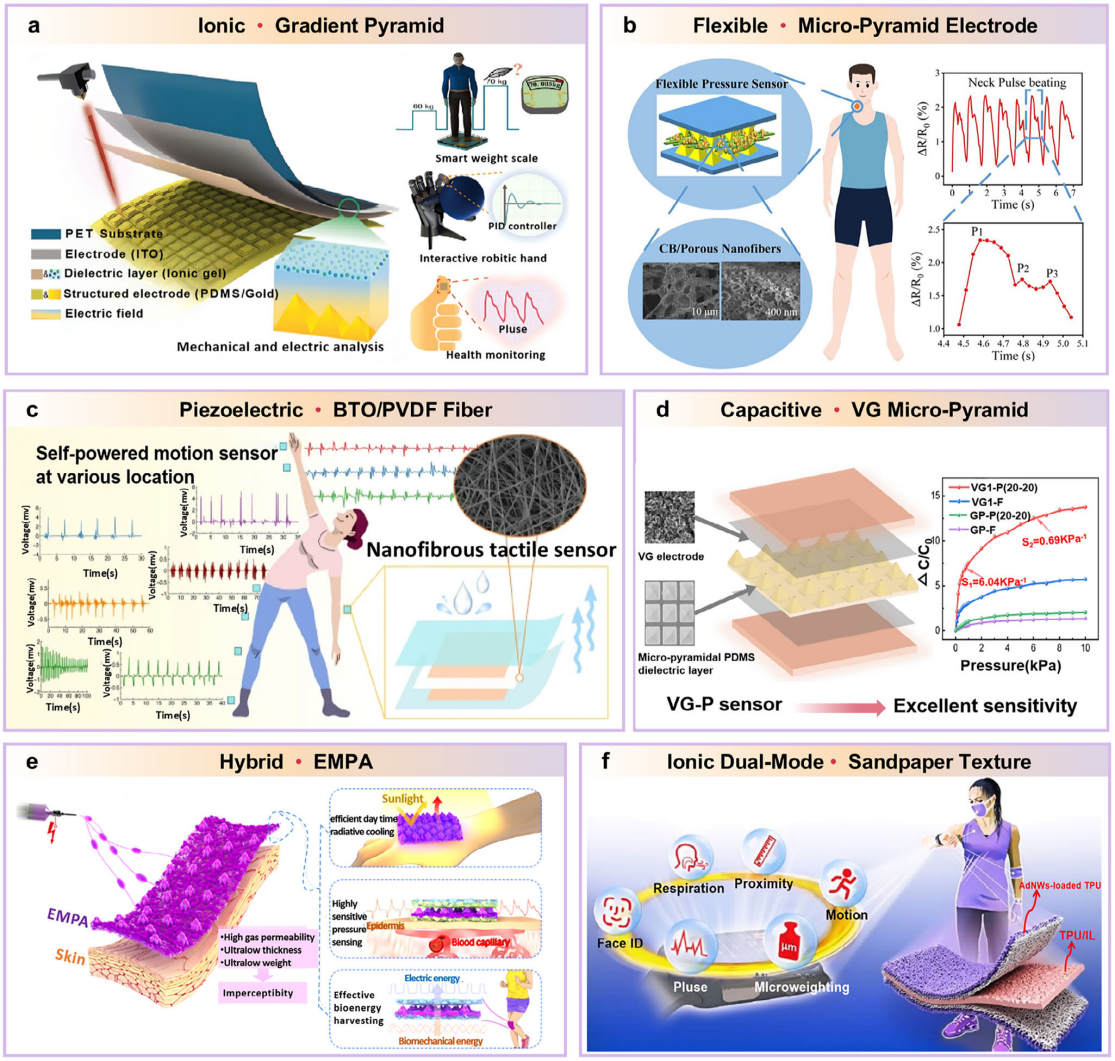
a) An ionic electronic pressure sensor‐based design schematic. Adapted with permission.^[^ ^118^ ^]^ Copyright 2023, Springer Nature. b) Pressure sensor preparation procedure using porous nanofibers' flexibility.Reproduced with permission.^[^ ^107^ ^]^ Copyright 2024, Elsevier. c) Lead‐free composite fibers provide self‐powered wearable sensors for human detection. Reproduced with permission.^[^ ^117^ ^]^ Copyright 2023, Springer. d) The procedure for making flexible capacitive pressure sensors. Adapted under the terms of the CC‐BY 4.0 license.^[^ ^119^ ^]^ Copyright 2023, The Authors. e) EMPA preparation, structure, and function based on multifunctional self‐assembled electrospinning micropyramidal arrays. Reproduced with permission.^[^ ^120^ ^]^ Copyright 2022, Springer Nature. f) Structure of a sandpaper‐shaped ionic piezoelectric sensor.Reproduced with permission.^[^ ^112^ ^]^ Copyright 2023, Elsevier.

The performance of the sensor is critically influenced by the choice of structural design and preparation techniques. Thorough optimization of the material structure system enhances the sensor's effectiveness and expands its applicability across diverse domains. To this end, **Table** **2** systematically summarizes and compares the performance enhancements in flexible pressure sensors achieved through different material and structural innovations. **Figure** **5**a illustrates the development of an ionic pressure sensor featuring a gradient pyramid microstructure, fabricated through programmable microstructure methodologies.^[^ ^102^ ^]^ This sensor, recognized for its remarkable response time and recovery performance, is applicable in robotic manipulators, weighing scales, and health monitoring systems. The enhanced sensor achieves a linear range of 1700 kPa, a detection limit of 0.36 Pa, and a sensitivity of 33.7 kPa^−1^. Owing to its high sensitivity, this sensor is well‐suited for applications demanding precise measurements and detection of minute pressure variations.

**Table 2:** Comparison of performance optimization effects of different materials/structural innovations on flexible pressure sensors.

Material	Structure	Mechanism	Sensitivity [kPa^−1^]	Detection range [kPa^−1^]	Cyclic stability	LOD [Pa]	Response/recovery [ms]	References
TPU / Ionic liquid	programmable	EDL‐enhanced Capacitive	37347.98	0–151.6	1500	3.54	111/66	[103]
			130.93–1400.49	0–956.7		7.08	20/40	
PVA/H_3_PO_4_	Fabric Microstructure	EDL‐enhanced Capacitive	242	0–1000	11000	–	52/13	[104]
MXene/PDMS	cylindrical microstructure	Piezoresistive	590	0–80	500	–	62.7/ 62.8	[101]
MXene/WPU/PU sponge	homogeneous interfacial locking	Piezoresistive	6226	0–146	100000	–	50/27	[105]
AgNWs/PDMS	gradient wrinkle structure	Piezoresistive	0.947	0.01–50	3000	10	80/115	[106]
PVDF‐HFP	micro‐pyramid	EDL‐enhanced Capacitive	33.7	0–1700	4500	0.36	6/11	[102]
PLA/PDMA	Porous and micro‐pyramid	Capacitive/ hybrid	54.06	0–56	10000	2.5	–	[107]
PDMS/PEDOT:PSS	Random‐height micro‐pyramid	Piezoresistive	391	0–2.58	150	350	52.91/ 4.38	[108]
ATMP‐PVA/Au‐PDMS	micro‐pyramid	EDL‐enhanced Capacitive	700	0–800	250	50	30/–	[109]
PVDF/TPU/PVA+Au	micro‐pyramid	Piezoelectric‐Friction Hybrid	19	0.05–	1000	0.05	48/– <0.8ms (triboelectric)	[110]
LIG electrode + PVA/H_3_PO_4_ ionic gel	3D porous structure	EDL‐enhanced capacitive	986.8	>200	2500	–	10/16	[111]
TPU/IL+ AgNWs‐TPU	3D mesh structure	EDL‐enhanced capacitive	106.01	0–77	2000	1.18	16/25	[112]
MXene/polyester textile + TPU/Ag	3D porous structure	Piezoresistive	652.1	0–60	8000	1	36/20	[113]
RGO‐coated PU Sponge	3D Porous structure	Piezoresistive	17.65	0–30.4 Strain: 0–80%	8000	–	–	[114]
Liquid Metal/TPU/MXene	3D Porous structure	Capacitive	1.91	0.01–260	4000	10	60/110	[115]

In recent years, rapid advancements in new material technologies and nanomaterial fabrication techniques have unlocked innovative opportunities for the design and application of sensors. Notably, the development of 3D materials, such as porous carbon structures and vertical graphene (VG), has paved the way for novel sensor architectures. Figure 5b depicts a flexible capacitive pressure sensor utilizing VG integrated with micro‐pyramidal structures.^[^ ^116^ ^]^ The integration of VG electrodes with polydimethylsiloxane (PDMS)‐based micro‐pyramidal dielectric layers facilitates significantly improved sensitivity for signal detection. Under applied pressure, the microstructured PDMS dielectric layer undergoes marked localized deformation, resulting in a substantial increase in capacitance variation.

A flexible pressure sensor designed to monitor various physiological parameters of the human body has been developed with a “sandwich” structure, constructed using electrospinning and abrasive transfer methods, as shown in Figure 5c. The electrodes consist of a PDMS micro‐pyramidal array film, whereas the sensitive layer is composed of a polylactic acid (PLA) fibrous film.^[^ ^107^ ^]^ The device incorporates a porous nanofiber network membrane and a micro‐pyramidal architecture. Exhibiting remarkable performance, the sensor achieves a sensitivity of 54 060 Pa^−1^ after 10 000 cycles and is capable of detecting a wide range of pressures from 0 to 56 000 Pa. Self‐assembled multifunctional electrospun micro‐pyramidal arrays (EMPAs), as shown in Figure 5d, represent a hybrid sensor that integrates both piezoelectric and triboelectric effects within a breathable, thin, and lightweight structure.^[^ ^110^ ^]^ These sensors exhibit excellent suitability for a broad range of applications, including energy harvesting, health diagnostics, and daytime radiation cooling.

Piezocapacitor–triboelectric hybrid sensors based on EMPAs demonstrate a rapid response time of under 0.8 ms and an ultralow detection limit of 0.05 Pa. By optimizing EMPA architectures and materials, the integration of triboelectric nanogenerators (TENGs) and PENGs significantly enhances the output voltage, optimizing device functionality and broadening their applicability across various domains. To enhance piezoelectric performance, the study regulated the β‐phase content of BaTiO_3_ nanoparticles within the PVDF matrix to exceed 80%, culminating in the development of a BaTiO_3_/PVDF nanofiber sensor, as illustrated in Figure 5e. By integrating triboelectric and piezoelectric effects, the device is capable of detecting multidimensional mechanical motion without the need for an external power source.^[^ ^117^ ^]^ Additionally, it produces electrical signals in reaction to external mechanical stimuli such as pressure, sliding, and vibration.

Figure 5f illustrates the structural configuration of a flexible ionic sensor that simultaneously detects pressure and temperature.^[^ ^112^ ^]^ The sensing mechanism relies on the elastic deformation of the sandpaper‐textured top layer, composed of thermoplastic polyurethane and ionic liquid (TPU/IL), which compresses the spacing between the upper and lower electrodes and enlarges the contact area with the intermediate electrode, thereby enhancing capacitance variation and facilitating signal transduction. As a temperature sensor, the device operates across a range of −40 to 80°C, with a temperature measurement accuracy of 0.02°C. When functioning as a pressure sensor, it offers a measurement range from 0 to 77 000 Pa, exhibiting a precision of 1.18 Pa. The sensor is capable of detecting subtle physiological signals, including swallowing, pulse, and facial expressions, alongside ambient temperature fluctuations, thus offering critical utility in health monitoring systems and human–machine interface technologies.

These results underscore its potential across a wide range of applications, particularly in wearable sensors and portable energy systems. In conclusion, the advancements in PENG‐based pressure sensors, along with the integration of self‐powered capacitors and piezoelectric‐driven energy storage systems, demonstrate their transformative capacity in overcoming the limitations inherent in conventional pressure sensors. These innovations lay the foundation for the development of next‐generation wearable electronics and IoT systems with enhanced sensitivity, durability, and energy efficiency.

This section examines the applications of PENGs in pressure sensing and biomechanical monitoring. Innovations in materials and microstructures, including micropyramids, ionogels, 3D networks, multilayer composites, and micro‐ and nanotextures, have substantially improved mechanical compliance and sensing accuracy. Future research should prioritize the development of multichannel signal‐fusion and dynamic‐calibration frameworks that integrate deep‐learning algorithms, thereby enabling high‐precision, self‐powered monitoring systems for wearable health tracking and motion analysis.

### 3.3 Biomedical Diagnostics and Health Monitoring

#### 3.3.1 Skin‐Interfaced Biosensing Platforms

Biosensors enable real‐time monitoring of physiological signals by integrating signal processing systems with advanced technologies, which enables early detection and prevention of illnesses.^[^ ^121^ ^]^ The growing awareness of global health concerns, coupled with rapid advancements in sensing technology, has significantly broadened the application scope of biosensors. However, despite their advantages, such as low cost, rapid response, and high sensitivity, traditional biosensors encounter intrinsic limitations, including environmental sensitivity and the short lifespan of bio‐elements.

As the largest organ in the human body, the skin serves as an ideal platform for biosensors due to its flexibility, biocompatibility, and self‐healing properties. When integrated with conductive nanomaterials, skin‐based biosensors can detect biomolecules such as enzymes, urea, and glucose, thus simulating the sensory functions of natural skin.^[^ ^122^, ^123^ ^]^ Self‐powered biosensors harvest mechanical energy generated by human movement or environmental stimuli and convert it into electrical energy to maintain device operation. These devices, including electronic skin (e‐skin) systems, can also store energy, which enhances device stability and service life.

**Figure 6:**
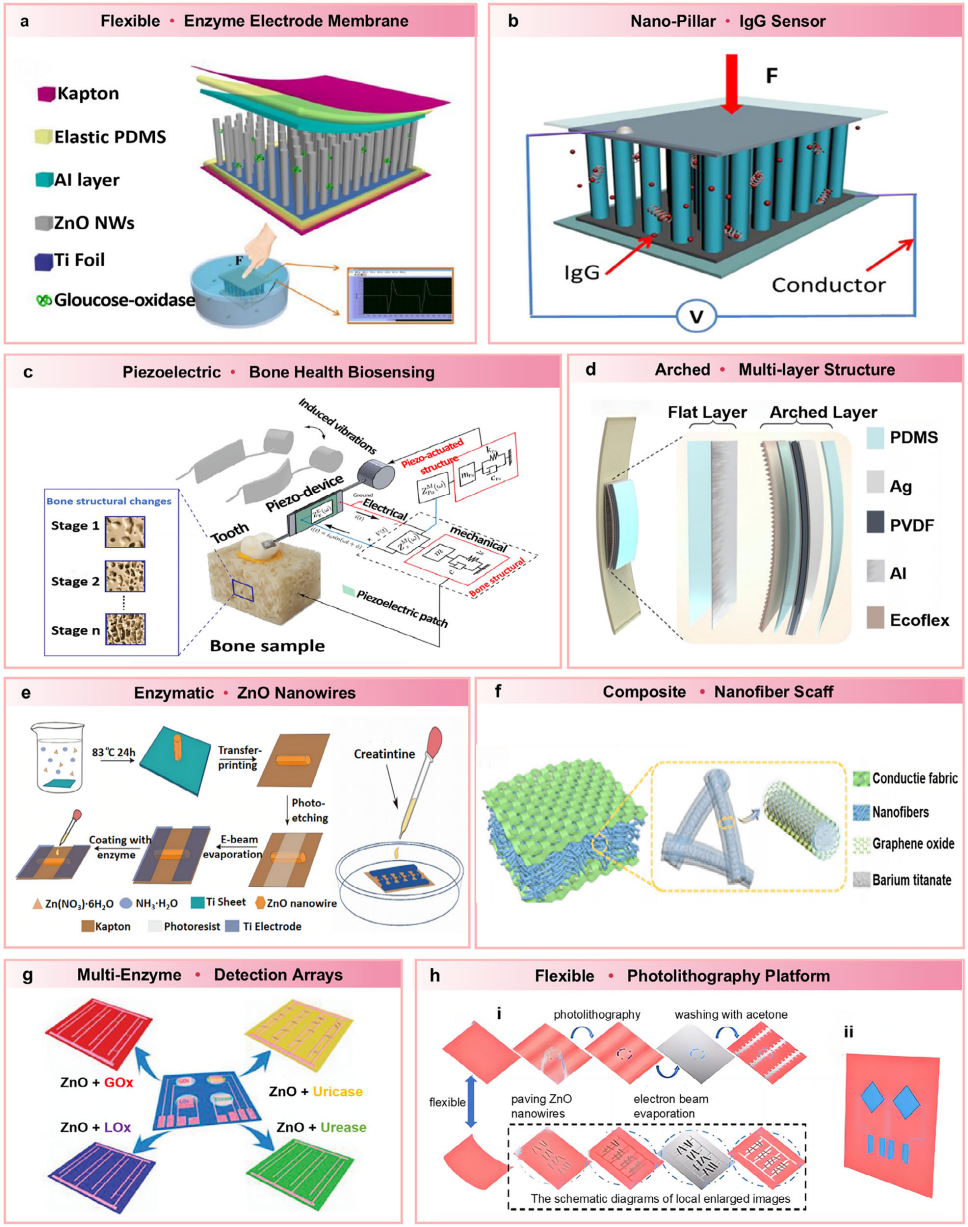
a) Structural layout of the e‐skin that runs on its own power. The gadget can be operated by human motion and in a liquid solution. Reproduced with permission.^[^ ^52^ ^]^ Copyright 2016, Elsevier. b) Diagram illustrating the self‐powered IgG detection system. Reproduced with permission.^[^ ^124^ ^]^ Copyright 2014, Elsevier. c) Monitoring of changes in bone structure using a piezo‐device and the EMI approach. Reproduced with permission.^[^ ^125^ ^]^ Copyright 2020, Springer. d) The chemical diagram illustrates the structure of SANG. Reproduced with permission.^[^ ^126^ ^]^ Copyright 2020, Elsevier. e) The self‐powered creatinine biosensor's fabrication. Reproduced under the terms of the CC‐BY 4.0 license.^[^ ^127^ ^]^ Copyright 2021, the authors. f) A schematic representation of a single electronic skin unit. A close‐up of the piezoelectric nanofibers' core and shell. Reproduced with permission.^[^ ^128^ ^]^ Copyright 2020, Elsevier. g) LOx, GOx, uricase, and urease are used to modify the piezo‐biosensing units in the electronic skin, accordingly. Reproduced with permission.^[^ ^129^ ^]^ Copyright 2017, American Chemical Society. h) Material system and device architecture, and two biosensor units are included in the test e‐skin to detect urea and uric acid independently. Reproduced with permission.^[^ ^130^ ^]^ Copyright 2017, The Royal Society of Chemistry.

**Figure** **6**a illustrates a self‐powered biosensor proposed by Xue et al. (2016), which operates based on a piezoelectric–enzyme‐reaction coupling mechanism.^[^ ^52^ ^]^ The device, supported by Kapton plates, generates electrical pulses when a human finger applies pressure to a liquid solution. It incorporates titanium foil and elastic PDMS‐Al electrodes. Another example, shown in Figure 6b, involves a biosensor powered by nanoelectric generation based on ZnO nanowires. After immersion in an IgG solution followed by rinsing and drying, the device converts external pressure into piezoelectric output for energy storage and power supply.^[^ ^124^ ^]^ Figure 6c depicts a piezoelectric‐actuated sensor integrated with an electromagnetic interference (EMI)‐based piezoelectric system for assessing bone density. Mechanical vibrations sensed by piezoelectric elements are transformed into electrical signals, enabling the detection of changes in bone structure under tension.^[^ ^125^ ^]^ Figure 6d presents a battery‐powered biosensor with an arched‐planar bilayer composite structure exhibiting anti‐biofouling capabilities.^[^ ^126^ ^]^ This sensor comprises two functional layers made from PDMS, Ecoflex, and PVDF. By adjusting the mass ratio between Ecoflex and PDMS, the curvature of the structure can be tuned. A stress gradient formed between the materials ensures consistent contact with body joints such as the elbow and wrist during motion. Through the synergistic integration of piezoelectric and triboelectric mechanisms, the sensor converts mechanical stimuli into electrical signals, allowing for real‐time monitoring of pulse signals.

#### 3.3.2 Metabolite Detection and Disease Diagnosis

For detecting early indicators of uremia, such as urea nitrogen and creatinine, traditional methods are often constrained by device size and power requirements. As illustrated in Figure 6e, researchers developed a self‐powered creatinine biosensor based on enzyme‐modified zinc oxide nanowires. The ZnO nanowires were synthesized using immersion and photolithography techniques, followed by the deposition of Ti electrodes through electron‐beam evaporation.^[^ ^127^ ^]^ Enzymes such as creatine oxidase, creatininase, and creatinase were incorporated to complete the biosensor assembly. This self‐powered device offers a compact and efficient platform for creatinine detection. Figure 6f presents a self‐sustained electronic skin (e‐skin) system with a core–shell structural configuration.^[^ ^128^ ^]^ The conductive layer, formed by a 3D interwoven network of silver nanowires, captures electromyographic signals, while the piezoelectric layer responds to pressure induced by muscle contractions. A graphene oxide‐based biointerface converts these signals into measurable electrical currents via enzymatic catalysis of the urea/lactate electrochemical reaction, thereby facilitating synergistic feedback across the three integrated functional layers.

Figure 6g illustrates a configuration comprising four distinct piezoelectric biosensing units, each functionalized with urease, glucose oxidase (GOx), lactate oxidase (LOx), and uricase, all immobilized on ZnO nanowires.^[^ ^129^ ^]^ During enzymatic catalysis, molecular charge alterations are transduced into electrical signals, thereby enabling autonomous and selective detection of multiple sweat metabolites, including lactic acid, glucose, uric acid, and urea. Figure 6h illustrates a self‐powered, implantable electronic skin (e‐skin) designed for real‐time in situ monitoring of urea and uric acid to support the diagnosis of renal disorders.^[^ ^130^ ^]^ The first layer in Figure 6h(i) outlines the structural configuration, fabrication methodology, and material stacking process. Initially, the main sensing layer is created by uniformly applying an appropriate quantity of ZnO onto a Kapton substrate. The desired pattern is then defined via photolithography. To enhance conductivity, a conductive layer is precisely deposited through an electron‐beam evaporation process. Acetone solution is subsequently applied and rinsed to finalize the micro‐pattern. The second layer functions as a flexible substrate, providing detailed local structural magnification of the e‐skin. Dual biosensing units, shown in Figure 6h(ii), can operate independently for simultaneous detection. Each unit is capable of identifying multiple biomolecules concurrently, allowing for parallel multi‐analyte detection and significantly improving the efficiency and comprehensiveness of daily health monitoring. The total device area measures ≈10 mm^2^.

#### 3.3.3 Multifunctional Wearable Health Monitors

**Figure 7:**
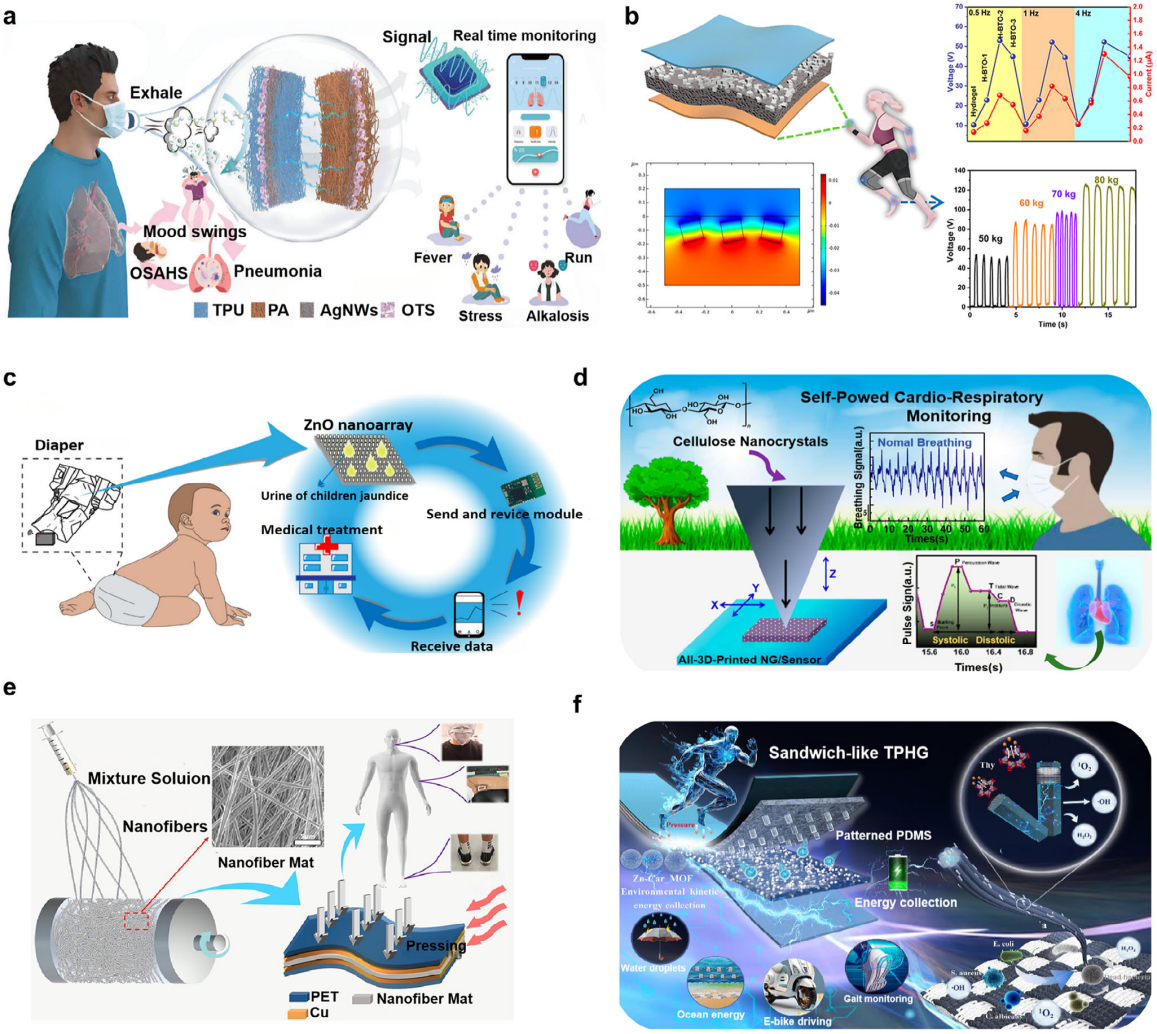
a) Multifunctional ASRS sensors can be used on masks to monitor respiratory health. Reproduced with permission.^[^ ^131^ ^]^ Copyright 2024, Wiley‐VCH. b) Stretchable multimodal sensor using asymmetric piezoelectric BaTiO_3_composite hydrogel. Reproduced with permission.^[^ ^132^ ^]^ Copyright 2022, American Chemical Society. c) Self‐powered wearable biosensor in baby diapers to detect newborn jaundice. Reproduced under the terms of the CC‐BY 4.0 license.^[^ ^133^ ^]^ Copyright 2022, the authors. d) Fully 3D‐printed cellulose‐based piezoelectric nanogenerator allows for self‐powered cardiopulmonary monitoring. Reproduced with permission.^[^ ^134^ ^]^ Copyright 2023, American Chemical Society. e) PAN/TMAB composite fiber felt‐based flexible nanogenerator for self‐powered multifunctional sensor applications. Reproduced with permission.^[^ ^135^ ^]^ Copyright 2022, American Chemical Society. f) TPHG combines with MOF and fibers to create self‐powered wearable medical devices. Reproduced with permission.^[^ ^136^ ^]^ Copyright 2025, Wiley‐VCH.

Wearable technology is capable of monitoring various physiological parameters, including skin pH, humidity, and lactate levels. Additionally, sweat on the skin contributes to improved metabolism by facilitating toxin removal and thermoregulation. As illustrated in **Figure** **7**a, the sensor integrates an octadecyltrichlorosilane (OTS)‐induced hydrophobic treatment, a silver nanowire (Ag NW) conductive framework, and TPU/PA composite layers, forming a multilayer autonomous sensing system (ASRS).^[^ ^131^ ^]^ This system exhibits excellent hydrophobicity and dependable sensing performance, enabling real‐time monitoring of respiratory activity, body movement, demographic traits (e.g., gender and age), and conditions such as sleep apnea.

An asymmetric composite film comprising barium titanate (BaTiO_3_) and polyacrylamide hydrogel (PAM/BTO) is depicted in Figure 7b. To enhance the efficiency of charge generation and storage, the design strategically optimizes the distribution and concentration of BTO, while leveraging the hydrogel's elasticity and the inherent piezoelectric properties of BTO. These combined features significantly enhance the sensor's sensitivity, facilitating precise detection of biomechanical cues such as curvature, pressure, and body motion.^[^ ^132^ ^]^

Ning et al.^[^ ^133^ ^]^ proposed a portable biosensor capable of detecting bilirubin and its oxidase without requiring an external power source, as shown in Figure 7c. Upon exposure to urine, the ZnO nanoarray facilitates free electron movement and charge transfer. The resulting voltage output, influenced by sodium bilirubin concentration and molecular dynamics, allows for accurate evaluation of neonatal health through enzymatic surface reactions involving bilirubin and bilirubin oxidase.

Figure 7d illustrates a fully 3D‐printed pyroelectric nanogenerator (Py‐PNG) capable of harvesting both mechanical and thermal energy, designed for self‐powered cardiopulmonary monitoring in wearable devices.^[^ ^134^ ^]^ The system integrates electrodes and piezoelectric components by leveraging a hydrogen‐bonded molecular network of biodegradable cellulose nanocrystals (CNC) through a layer‐by‐layer printing technique. By combining piezoelectric and pyroelectric functionalities, the device converts mechanical stimuli—such as pulse waves—and temperature fluctuations from respiration into electrical energy, enabling autonomous operation and broadening its applicability in real‐time health monitoring.

An electrospun piezoelectric and thermoelectric nanogenerator is presented in Figure 7e. The sensor comprises a composite nanofiber mat consisting of polyacrylonitrile (PAN) embedded with trimethylamine borane (TMAB), which enables dual‐mode sensing of both mechanical forces and thermal variations.^[^ ^135^ ^]^ This dual‐output capability overcomes the inherent limitation of conventional piezoelectric devices in detecting thermal stimuli. The system's precision in tracking respiratory dynamics and joint movement angles highlights its potential as a battery‐free platform for next‐generation wearable medical technologies.

Figure 7f showcases a piezoelectric‐friction hybrid nanogenerator designed using two types of bio‐metal–organic frameworks (Bio‐MOFs) for multifunctional applications. The top friction layer, composed of patterned micro‐conical PDMS, increases pressure sensitivity by expanding the contact surface under compression. The middle layer utilizes Zn‐Car‐MOF material to convert environmental kinetic energy—such as ocean waves and raindrops—into storable electrical energy via electrochemical means.^[^ ^136^ ^]^ The bottom layer integrates bactericidal Cu‐HHTP_MOF loaded with thymol (Thy), which neutralizes ROS with over 98% antimicrobial efficiency. This integrated design redefines the design paradigm of wearable biosensors by unifying energy harvesting, multimodal biosensing, and active antimicrobial defense into a single platform.

PENGs have demonstrated considerable versatility in biomedical monitoring, ranging from skin‐interfaced physiological signals to metabolite detection in biofluids. The integration of flexible materials and multimodal sensing strategies enhances both signal stability and wearer comfort. The most promising technical pathway is the integration of PENGs with microfluidic platforms, AI‐enabled signal processing, and wireless communication, thereby enabling autonomous smart medical devices and providing a pathway toward personalized healthcare.

### 3.4 Environmental Gas Sensing and Hazard Detection

#### 3.4.1 Principles of Self‐Powered Gas Sensors

Given the escalating threats to human health and ecosystems posed by the continuous emission of hazardous gases from various industrial activities, along with the increasing need for indoor air quality monitoring to enhance living standards,^[^ ^137^, ^138^ ^]^ gas sensors have emerged as promising tools for addressing these challenges. Despite the advantages of traditional metal oxide‐based gas sensors—such as low cost, compact size, and ease of fabrication—their high operating temperatures and dependence on external power sources limit their suitability for integration into IoT and wearable environmental monitoring systems.^[^ ^139^ ^]^

ZnO 1D nanostructures are considered highly sensitive and responsive gas‐sensing materials due to their high surface‐to‐volume ratio and superior surface adsorption properties, which increase interaction sites and accelerate gas molecule diffusion.^[^ ^140^ ^]^ Moreover, their intrinsic piezoelectric characteristics allow for the harvesting of environmental mechanical energy to generate sensing signals, effectively circumventing the limitations posed by conventional power sources. Specifically, the adsorption of gas molecules on the surface of piezoelectric semiconductor nanomaterials like ZnO alters the density of free charge carriers, thereby modulating the piezoelectric voltage output under mechanical stimulation—a phenomenon known as the screening effect. **Table** **3** summarizes the key properties of PENG‐based self‐powered gas sensors.

**Table 3:** A summary of PENG‐based self‐powered gas sensors.

Materials	Gas	Detection range	Concentration	Sensitivity		References
				a	b	
ZnO Nanowires	H_2_S	100–1000 ppm	1000 ppm	56	123.7	[17]
Cu/ZnO Nanowires	H_2_S	100–1000 ppm	1000 ppm	91.1	1045.8	[141]
NiO/ZnO Nanowires	H_2_S	100–1000 ppm	1000 ppm	84.3	536.1	[142]
CuO/ZnO Nanowires	H_2_S	200–800 ppm	800 ppm	86.3	629.8	[143]
CuO/ZnO Nanowires	H_2_S	100–500 ppm	500 ppm	382.4	79.3	[144]
In_2_O_3_/ZnO Nanowires	H_2_S	100–700 ppm	700 ppm	90.2	925	[145]
CdS Nanorods	H_2_S	200–600 ppm	600 ppm	62.5	166.7	[146]
CeO_2_/ZnO Nanowires	Humidity	5–95% RH	95% RH	82.1	457.1	[147]
Cd/ZnO Nanowires	Humidity	20–70% RH	70% RH	85.7	600	[148]
Fe/ZnO Nanowires	Humidity	5–60% RH	60% RH	75.3	304	[149]
Co/ZnO Nanowires	Humidity	20–70% RH	70% RH	82	454.7	[150]
SnO_2_/ZnO Nanowires	Humidity	5–85% RH	85% RH	76.4	323.1	[147]
ZnO Nanowires	Humidity	25–90% RH	90% RH	87.8	716.7	[144]
ZnO Nanowires	Ethanol	100–700 ppm	700 ppm	85	566.7	[151]
Pt/ZnO Nanowires	Ethanol	400–1500 ppm	1500 ppm	37.7	60.4	[152]
Au/ZnO Nanowires	Ethanol	400–1200 ppm	1200 ppm	72.1	258.1	[153]
Pd/ZnO Nanowires	Ethanol	200–800 ppm	800 ppm	51.9	108	[154]
Pd/ZnO Nanowires	Ethanol	200–1000 ppm	1000 ppm	59.8	148.9	[144]
Ag/ZnO Nanowires	Ethanol	10–1000 ppm	1000 ppm	90	900	[155]
α‐Fe_2_O_3_/ZnO Nanowires	Ethanol	100–700 ppm	700 ppm	87.6	706.8	[156]
SnO_2_/ZnO Nanowires	H_2_	200–800 ppm	800 ppm	82.5	471.4	[157]
ZnSnO_3_/ZnO Nanowires	LPG	1000–8000 ppm	1000 ppm	24.2	498.9	[158]
TiO_2_/ZnO Nanowires	CH_4_	100–500 ppm	500 ppm	87.5	46.7	[144]

^a)^ S % = (V_a_−V_g_)/V_a_ ×100 %. ^b^ S % = (V_a_−V_g_)/V_g_ × 100 %, where V_a_ and V_g_ are the output voltages of the device under the same conditions in dry air and the test gas, respectively;

#### 3.4.2 Hazardous Gas Detection Systems

**Figure 8:**
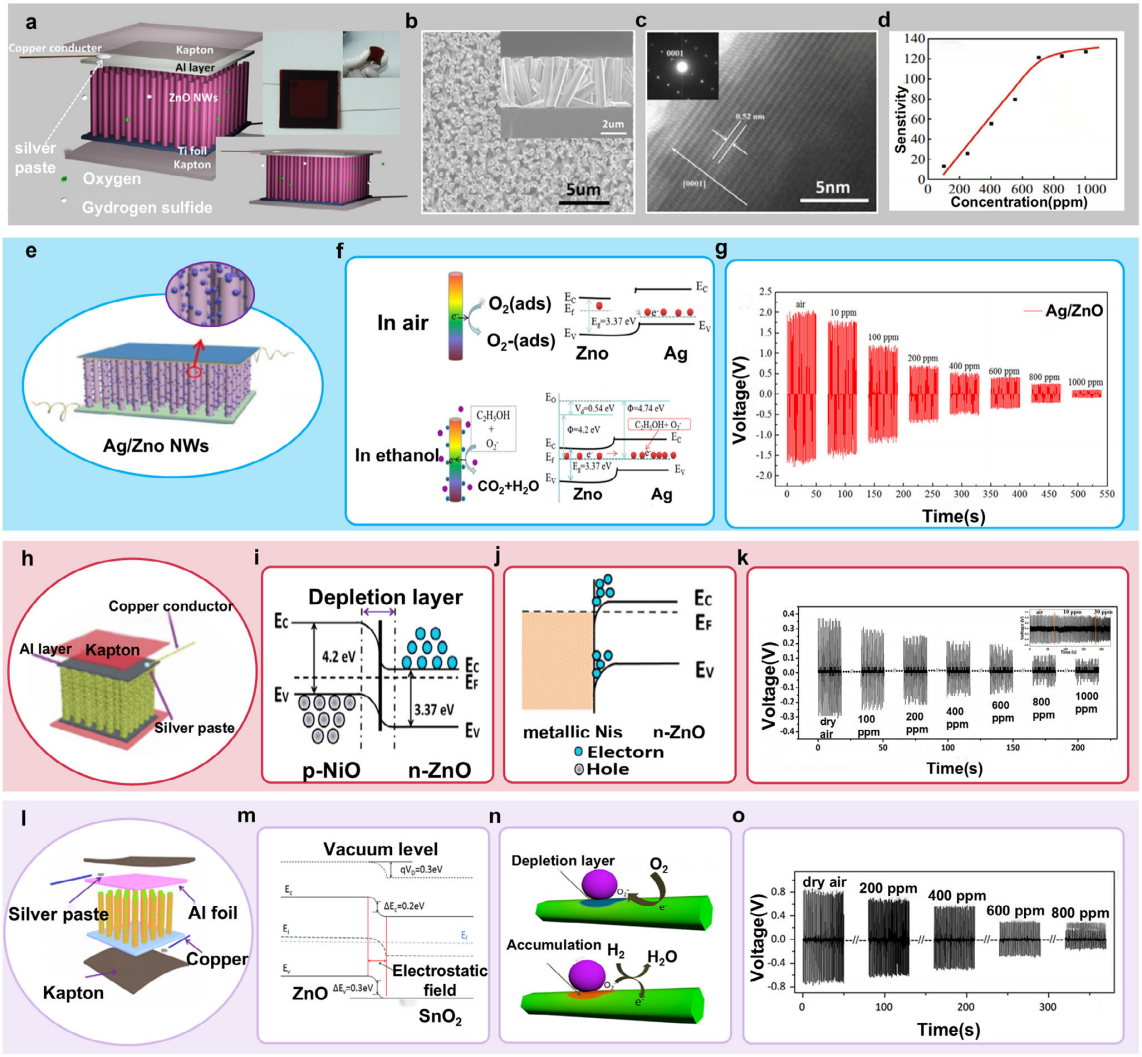
a) Schematic illustration of the structure of the first self‐powered active H_2_S gas sensor based on PENG (the inset is a photograph of a typical device). b) SEM image of ZnO NW arrays grown on Ti foil in a top view (the inset shows the SEM cross‐sectional view image). c) HRTEM and SAED pattern taken from the tip region of a ZnO NW. d) The dependence of sensitivity on the ranging concentrations of H_2_S vapor.Reproduced with permission.^[^ ^17^ ^]^ Copyright 2012, IOP Publishing. e) Schematic illustration of the structure of the Ag/ZnO‐based active ethanol gas sensor. f) The sensing mechanism of Ag/ZnO NWs for detecting ethanol. g) The piezoelectric output voltage of the device under the same applied compressive deformation at room temperature in dry air and various concentrations of ethanol.Reproduced with permission.^[^ ^155^ ^]^ Copyright 2021, Springer. h) Schematic illustration of the structure of the NiO/ZnO‐based active H_2_S gas sensor. i) The band diagram of the NiO/ZnO heterojunction. j) Band diagram of NiS/ZnO interface. k) The piezoelectric output voltage of the device under the same applied compressive deformation at room temperature in dry air and various concentrations of H_2_S (the inset shows the low detection limit). Reproduced with permission.^[^ ^142^ ^]^ Copyright 2015, Elsevier. l) Schematic illustration of the structure of the SnO_2_/ZnO‐based active H_2_ gas sensor. m) The band diagram of the SnO_2_/ZnO heterojunction. n) The sensing mechanism of SnO_2_/ZnO NWs for detecting H_2._ o) The piezoelectric output voltage of the device under the same applied compressive deformation at room temperature in dry air and various concentrations of H_2_. Reproduced with permission.^[^ ^157^ ^]^ Copyright 2014, Elsevier.

Xue et al.^[^ ^17^ ^]^ developed a self‐powered, active H_2_S gas sensor based on a PENG by integrating the piezoelectric and gas‐sensing properties of ZnO nanowires (NWs). As illustrated in **Figure** **8**a, the device structure comprises: i) titanium (Ti) foil serving as both the electrode and the growth substrate for ZnO NW arrays, ii) aluminum (Al) foil as the counter electrode, and iii) Kapton support frames. Figure  8b shows scanning electron microscopy (SEM) images of the top and cross‐sectional views of ZnO NW arrays synthesized via a wet chemical method, exhibiting average diameters of ≈500 nm and lengths of ≈5 µm. Figure  8c presents a high‐resolution transmission electron microscopy (HRTEM) image and the corresponding selected‐area electron diffraction (SAED) pattern of a ZnO NW tip, confirming structural uniformity, single crystallinity, and preferential *c*‐axis‐oriented growth. Benefiting from its well‐ordered architecture, the device demonstrated a wide H_2_S detection range (100–1000 ppm) and remarkable sensitivity, achieving a response of 127.3% at the highest tested concentration (Figure  8d).

Current research on PENG‐based self‐powered gas sensors is predominantly focused on ZnO. To date, various strategies—such as metal doping and heterostructure engineering—have been employed to enhance the sensitivity and selectivity of ZnO‐based gas sensors.^[^ ^159^ ^]^ He et al.^[^ ^155^ ^]^ developed a self‐powered ethanol gas sensor utilizing Ag‐doped ZnO nanowire (NW) arrays (Figure  8e). As illustrated in Figure  8f, the sensing mechanism involves band alignment between ZnO and Ag, which facilitates electron transfer from ZnO to Ag until Fermi level equilibrium is reached. This results in the formation of Schottky barriers at the ZnO/Ag interfaces. Due to Ag's superior oxygen dissociation capability compared with ZnO, more electrons are attracted to the ZnO surface to generate O_2_^−^ species under equilibrium conditions. When subjected to mechanical strain in an ethanol atmosphere, the higher surface electron density arising from Ag‐catalyzed ethanol dissociation produces a strong screening effect on the piezoelectric output. Consequently, increasing ethanol concentration leads to a gradual decrease in piezoelectric potential (Figure  8g).

Transition metals possess distinctive properties owing to their partially filled d‐orbitals.^[^ ^160^ ^]^ Incorporating transition metal ion dopants can effectively tune the band gap, electrical conductivity, defect states (donor/acceptor), and lattice parameters.^[^ ^161^ ^]^ Fu et al.^[^ ^141^ ^]^ reported a self‐powered, active H_2_S sensor achieved via Cu doping. The Cu dopants substitute for Zn sites in the ZnO lattice, creating donor defects that promote charge separation and transport. In addition, the synergistic interaction between Cu dopants and H_2_S molecules in Cu–ZnO NWs enhances H_2_S adsorption and its reaction with adsorbed oxygen species. This process increases carrier density while reducing piezoelectric output. As a result, the response and selectivity of the sensor were significantly enhanced compared with those of undoped ZnO devices.

Introducing heterojunctions at metal oxide interfaces can facilitate electron transfer and enhance oxygen adsorption, thereby improving the sensitivity and response speed of gas sensors. In p–n heterojunctions, electrons in the conduction band of the n‐type semiconductor migrate to the lower‐energy valence band of the p‐type semiconductor, resulting in a depletion layer at the interface due to electron–hole recombination.^[^ ^162^ ^]^ Qu et al.^[^ ^142^ ^]^ fabricated a NiO/ZnO‐based PENG gas sensor (Figure  8h). When ZnO nanowires (NWs) were coated with NiO nanoparticles via a wet chemical method, p–n heterojunctions formed between the two materials. This induced the formation of a charge depletion layer and reduced electron density in the ZnO NWs (Figure  8i). Upon exposure to H_2_S, NiO nanoparticles reacted with H_2_S, converting to NiS. This reaction destroyed the NiO/ZnO p–n junction and its depletion layer, transforming the contact into a NiS/ZnO Ohmic interface (Figure  8j). During this process, the electron density greatly increased due to the NiS/ZnO Ohmic contact, enhancing the screening effect and thereby reducing the piezoelectric output. As shown in Figure  8k, compared with 0.388 V in dry air, the piezoelectric voltage decreased from 0.295 V to 0.061 V as the H_2_S concentration increased.

In n–n heterojunctions, electrons migrate from the semiconductor with a higher Fermi level to that with a lower Fermi level. The resulting electron accumulation layer can be depleted through oxygen adsorption on the surface, increasing the potential barrier at the interface.^[^ ^162^ ^]^ Fu et al.^[^ ^157^ ^]^ developed a self‐powered active H_2_ sensor employing SnO_2_/ZnO n–n heterojunctions (Figure  8l). As shown in Figure  8m, electrons flow from SnO_2_ to ZnO until Fermi level equilibrium is reached, forming a depletion layer on the SnO_2_ side and an accumulation layer on the ZnO side. In dry air, oxygen molecules adsorb on the SnO_2_/ZnO surface, capturing free electrons provided by ZnO near the interface and forming chemisorbed oxygen ions. This reduces the free electron density in ZnO, resulting in a high piezoelectric output. Upon exposure to H_2_, electrons released by the oxidation reaction (O_2_^−^ + 2H_2_ → 2H_2_O + e^−^) return to the ZnO conduction band, significantly increasing the surface electron density and screening the piezoelectric field (Figure  8n). Consequently, the piezoelectric potential decreases markedly, while the sensor sensitivity increases (Figure  8o).

He et al.^[^ ^144^ ^]^ reported a flexible, self‐powered “electronic smelling skin” (e‐skin) constructed from a piezo‐gas‐sensor matrix composed of ZnO‐based composite nanowires (NWs) via a soft photolithography approach, designed for real‐time monitoring of mining environments with high sensitivity and selectivity. The e‐skin integrates four distinct ZnO‐based composite NW types—bare ZnO NWs, Pd/ZnO NWs, CuO/ZnO NWs, and TiO_2_/ZnO NWs—fabricated by hydrothermally depositing functional materials onto ZnO NW arrays, each serving as an individual piezo‐gas‐sensing unit. These units are capable of cross‐reactively detecting relative humidity (RH), ethanol, hydrogen sulfide (H_2_S), and methane (CH_4_) without the need for external power sources. The device exhibited response rates of 87.76% for 90% RH, 59.82% for 1000 ppm ethanol, 79.27% for 500 ppm H_2_S, and 87.50% for 500 ppm CH_4_. Notably, the e‐skin can be worn on a miner's body, where it continuously harvests mechanical energy to enable continuous, real‐time monitoring of the working environment.

#### 3.4.3 Advanced Architectures for Selective Sensing

For room‐temperature monitoring of liquefied petroleum gas (LPG)—a widely used gaseous fuel—Fu et al.^[^ ^158^ ^]^ employed the gas‐sensing capabilities of perovskite‐type ternary compounds (ABO_3_) by incorporating ZnSnO_3_/ZnO heterostructures into PENGs. In addition, PENG‐driven active gas sensors have been successfully utilized for detecting other hazardous gases, including hydrogen^[^ ^157^ ^]^ and nitrogen dioxide.^[^ ^163^ ^]^ Generally, these gas sensors leverage the dual functionality of piezoelectric semiconductors, which serve simultaneously as energy harvesters and sensing elements. However, the requirement that a single material possesses both piezoelectric and gas‐sensing capabilities restricts the range of applicable materials, thereby limiting the practicality and gas selectivity of such sensors.^[^ ^164^ ^]^

**Figure 9:**
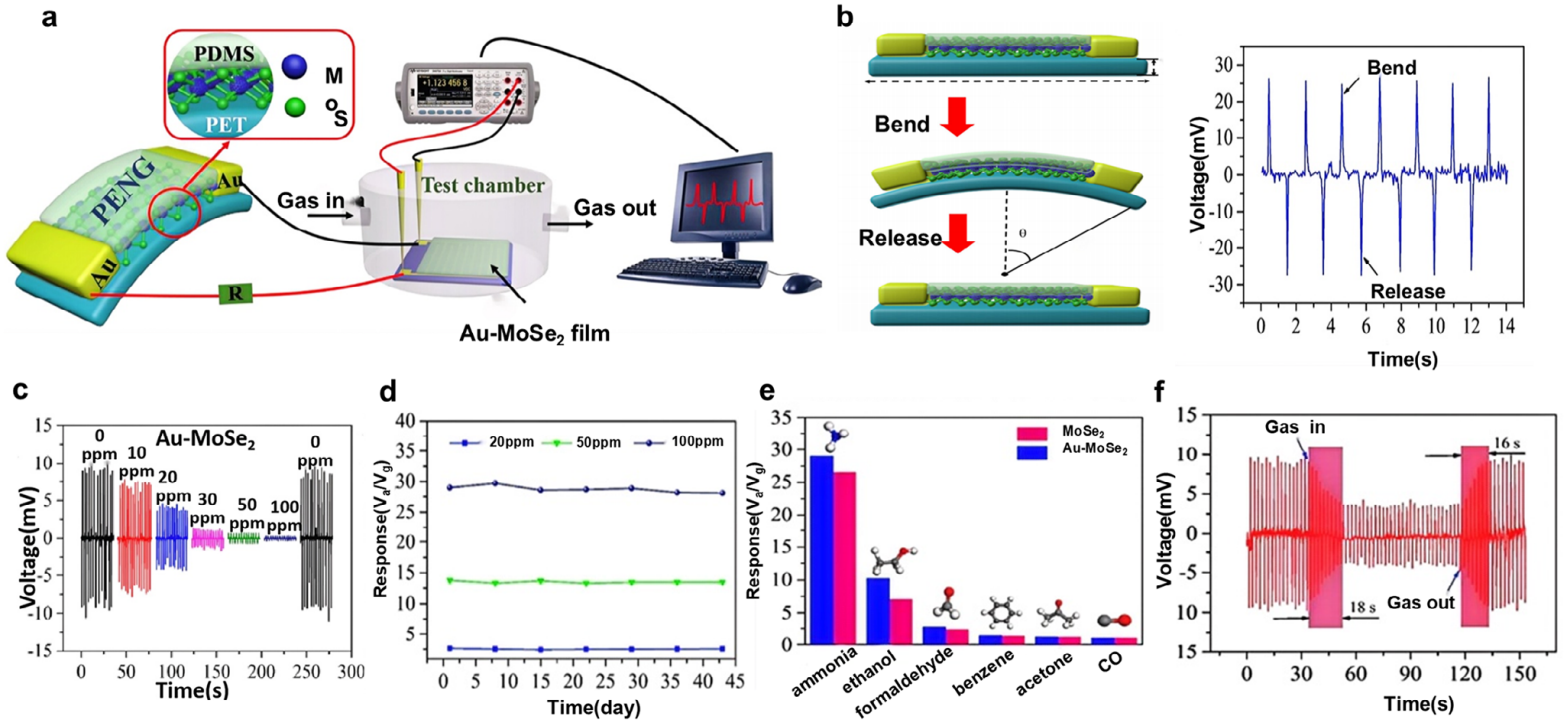
a) Schematic illustration of a self‐powered NH_3_ sensor driven by MoS_2_‐flake‐based PENG. b) Schematic diagram of the bending and releasing of the PENG device (the inset shows the output voltages of the device under constant strain of 0.36%). c) The piezoelectric output voltage of the Au‐MoSe_2_‐based gas sensor under the same applied compressive deformation at room temperature, for various concentrations of NH_3_. d) Long‐term stability test of Au‐MoSe_2_‐based gas sensor toward 20, 50, and 100 ppm of NH_3_ for 7 weeks. e) Selectivity of the Au‐MoSe_2_‐based gas sensor upon exposure to different interfering gases of 100 ppm. f) Dynamic response/recovery curve of the Au‐MoSe_2_‐based gas sensor to 20 ppm of NH_3_ at room temperature. Reproduced with permission.^[^ ^165^ ^]^ Copyright 2019, Elsevier.

To overcome these limitations, Zhang et al.^[^ ^165^ ^]^ introduced a flexible, room‐temperature ammonia (NH_3_) sensor using an Au‐functionalized MoSe_2_ composite, powered by a MoS_2_‐based PENG (**Figure** **9**a). The PENG utilizes monolayer MoS_2_ flakes as the piezoelectric layer, polyethylene terephthalate (PET) films as the substrate, PDMS as a protective layer, and Au electrodes, achieving efficient mechanical energy harvesting and robust piezoelectric performance. As shown in Figure 9b, the PENG produces a peak voltage output of 26 mV under a bending strain of 0.36% at a frequency of 0.5 Hz. Functionalization with Au nanoparticles (NPs) enhances NH_3_ detection by decreasing sensor resistance and accelerating molecular oxygen dissociation to create more active adsorption sites. As a result, the output voltage decreases significantly with increasing NH_3_ concentration (Figure 9c). The sensor demonstrates excellent long‐term stability, with less than 5% deviation and no apparent signal degradation over seven weeks of continuous exposure (Figure 9d). Its high selectivity toward NH_3_ at 100 ppm, compared to other interfering gases, is illustrated in Figure 9e. Rapid sensing dynamics are also achieved, with response and recovery times of 18 s and 16 s, respectively, for 20 ppm NH_3_ (Figure 9f). This work exemplifies the potential of integrating separate energy harvesting and sensing components to develop next‐generation self‐powered gas sensors.

Subsequent studies have extended this approach by introducing a poly(vinyl alcohol)/Ti_3_C_2_T_x_ (PVA/MXene)‐based humidity sensor powered by a MoSe_2_‐PENG,^[^ ^166^ ^]^ and a formaldehyde (HCHO) sensor based on an MXene/Co_3_O_4_ composite, driven by ZnO/MXene nanowire array‐based PENGs.^[^ ^167^ ^]^

Although advanced PENG‐based structures have demonstrated strong performance in detecting gases such as NH_3_ and formaldehyde, achieving selective detection and identification of light hydrocarbon gases (e.g., liquefied petroleum gas [LPG], methane, propane) remains a significant challenge. Because PENGs primarily function as mechanical‑to‑electrical energy converters, their piezoelectric materials inherently lack intrinsic chemical selectivity toward specific gas species. Moreover, hydrocarbon gases are chemically inert and possess highly similar molecular structures, resulting in minimal differences in adsorption behavior and charge‑transfer interactions at material surfaces. Consequently, variations in piezoelectric output are subtle and difficult to resolve. Ambient humidity and temperature further confound detection, rendering reliable sensing impractical when relying solely on the intrinsic selectivity of the piezoelectric material.^[^ ^168^ ^]^

To overcome the selectivity limitations of PENG‑based hydrocarbon‑gas sensing, recent research has shifted from device‑level optimization to system‑level integration strategies. First, incorporating metal nanoparticles (e.g., Pt, Pd, Ag) as catalytic sites can effectively modulate the adsorption and dissociation kinetics of gas molecules, thereby amplifying differential responses among various hydrocarbons. For example, a Pt/Mn_3_O_4_ sensor utilizes Pt‑induced activation of ethylene and exhibits a sensor response of 7.4 at 1000 ppm, enabling efficient discrimination of ethylene from methane and propane.^[^ ^169^ ^]^ Similarly, Ag‑modified ZnO has been shown to enhance both the response and selectivity toward CH_4_. Such catalyst‑modification strategies can be directly integrated with the piezoelectrically active layer in PENGs, thereby amplifying gas‑specific signatures in the PENG output.^[^ ^170^ ^]^

Second, porous and structured sensing layers can leverage diffusion dynamics and steric effects to accentuate subtle differences among hydrocarbons. Qin et al. developed a six‑channel MOF–QCM array capable of discriminating xylene isomers through differentiated adsorption (limit of detection: 1 ppm; classification accuracy in mixtures: 96.5%).^[^ ^171^ ^]^ Meanwhile, modulating the interlayer and intralayer spacing of two‑dimensional fluorinated MOFs (ZUL series) enables selective separation of acetylene and ethylene,^[^ ^172^ ^]^ providing design insights for engineering sensitive layers in PENG‑based sensors.^[^ ^173^ ^]^

Third, integrating multiple sensing materials that exhibit differential responses to various hydrocarbon gases and coupling them with machine‑learning models transforms multidimensional weak‑selectivity signals into robust identification outputs, thereby enabling the construction of intelligent sensing systems. In electronic‑nose research, algorithms such as PCA, SVM, random forests, and convolutional neural networks (CNNs) extract features from complex multichannel waveforms to achieve accurate classification of multicomponent hydrocarbon mixtures; feature‑extraction pipelines combined with appropriate classifiers further enable discrimination of complex gas mixtures.^[^ ^174^, ^175^ ^]^ Moreover, recent studies using MOS sensors combined with deep learning and MEMS gas sensors paired with lightweight algorithms such as MiniRocket (which enable efficient classification and concentration prediction for eight gases) provide transferable pathways for future self‑powered intelligent recognition systems driven by PENG output signals.^[^ ^176^, ^177^ ^]^

Within PENG‑based platforms, such integration offers the unique advantage of enabling the device to function simultaneously as a power source and as a gas‑signal transducer, thereby allowing on‑device (edge) algorithms to perform gas identification under ultralow‑power conditions. Future efforts should focus on establishing standardized datasets that encompass variations in temperature and humidity and common interfering gases, as well as developing robust, energy‑efficient, edge‑intelligent algorithms, to facilitate the translation of self‑powered hydrocarbon‑gas sensing systems from laboratory research to real‑world deployment.

Building on existing studies, PENG‑based gas sensors have achieved substantial progress toward self‑powered continuous monitoring. The synergistic design of composite materials and micro‑ and nanostructures has enhanced both response time and gas selectivity. Future research should focus on developing multimodal recognition mechanisms and sensor‑node networking technologies, integrated with AI algorithms, to enable accurate detection and early warning for multiple target gases in complex environments.

### 3.5 Toward Self‐Powered Intelligent Systems Enabled by PENGs

PENGs directly transduce mechanical deformation into electrical signals while offering high flexibility and stretchability. They are transitioning from simple energy‐harvesting devices to core components of self‐powered sensing and intelligent systems. Future systems are expected to integrate self‐powering, autonomous signal processing, multiphysics data fusion, and intelligent decision‐making capabilities. This section examines the development pathways of PENG‐driven intelligent systems from three perspectives: signal processing, system integration, and multimodal intelligence.

#### 3.5.1 AI‐Enhanced Signal Processing for PENG Sensors

With the rapid integration of artificial intelligence and nanogenerator technologies, PENGs have evolved from standalone energy‐harvesting units into intelligent nodes capable of self‐powering, sensing, and information interpretation. However, PENG signal outputs often depend on variations in surface adsorption states or transient mechanical stimuli. These signals are highly susceptible to environmental factors and surfacecharge fluctuations, frequently exhibiting low signal‐to‐noise ratios and blurred feature information. These limitations directly constrain the effectiveness of traditional feature extraction methods in complex scenarios such as human gesture recognition, hydrocarbon gas discrimination, and abnormal pulse detection.^[^ ^178^ ^]^

To address these limitations, recent studies have adopted lightweight models—such as CNNs, PCA, and LDA—to extract and filter features from generator output waveforms (e.g., peak amplitudes, rise times, and delays), thereby enabling precise processing and in‐depth analysis. This approach is a key strategy for enhancing the usability and reliability of PENG‐derived signals.^[^ ^179^ ^]^ For instance, Li et al. developed a flexible wearable device for knee joint motion monitoring.^[^ ^180^ ^]^ They applied the short‐time Fourier transform to extract frequencydomain features and combined them with a lightweight artificial neural network to continuously estimate knee torque and angle, achieving a ninelevel torqueclassification accuracy of 97.5%. Similarly, Yang et al. designed a TENG–PENG hybrid, self‐powered tactile sensor.^[^ ^181^ ^]^ Using a CNN with six convolutional layers and three pooling layers, they performed endtoend recognition directly on raw gesture signals, achieving an accuracy of 94.16% without manual feature engineering. These results convincingly demonstrate the critical role of AI in uncovering deep features in PENG signals.

In environmental monitoring, the integration of AI has significantly enhanced the gasdiscrimination capabilities of PENG‐based systems. Meng et al developed a ZnO‐based, temperaturemodulated gassensing array to capture dynamic response curves and employed a decision tree algorithm to identify four alcohol homologues.^[^ ^182^ ^]^ The system achieved a recognition accuracy of 97.62% at concentrations of 100–400 ppm, effectively addressing signal saturation and cross‐sensitivity among homologues, and thereby providing a reusable methodological framework for future PENGdriven gas sensing. Han et al. developed an intelligent system for sarcopenia screening.^[^ ^183^ ^]^ The system utilized a flexible, printed piezoelectric sensor array to collect plantar pressure signals, and supportvector machines (SVMs) were applied for feature analysis, achieving a screening accuracy of 93.65%. This approach offers a low‐cost, portable solution for early, community‐based screening.

Collectively, these studies demonstrate that integrating AI into PENG systems can improve reliability and generalizability in complex environments, thereby supporting the deployment of PENGs in intelligent wearables, environmental monitoring, auxiliary diagnostics, and next‐generation human–machine interaction.

#### 3.5.2 System‐Level Self‐Powered Architectures

In recent years, PENGs have evolved from standalone energy‐harvesting units into system‐level nodes that integrate sensing, power supply, and intelligent decision‐making. The core of such system architectures lies in the efficient coordination of energy harvesting, storage, management, and information processing, enabling sustainable, low‐power, continuous operation.^[^ ^184^ ^]^

**Figure 10:**
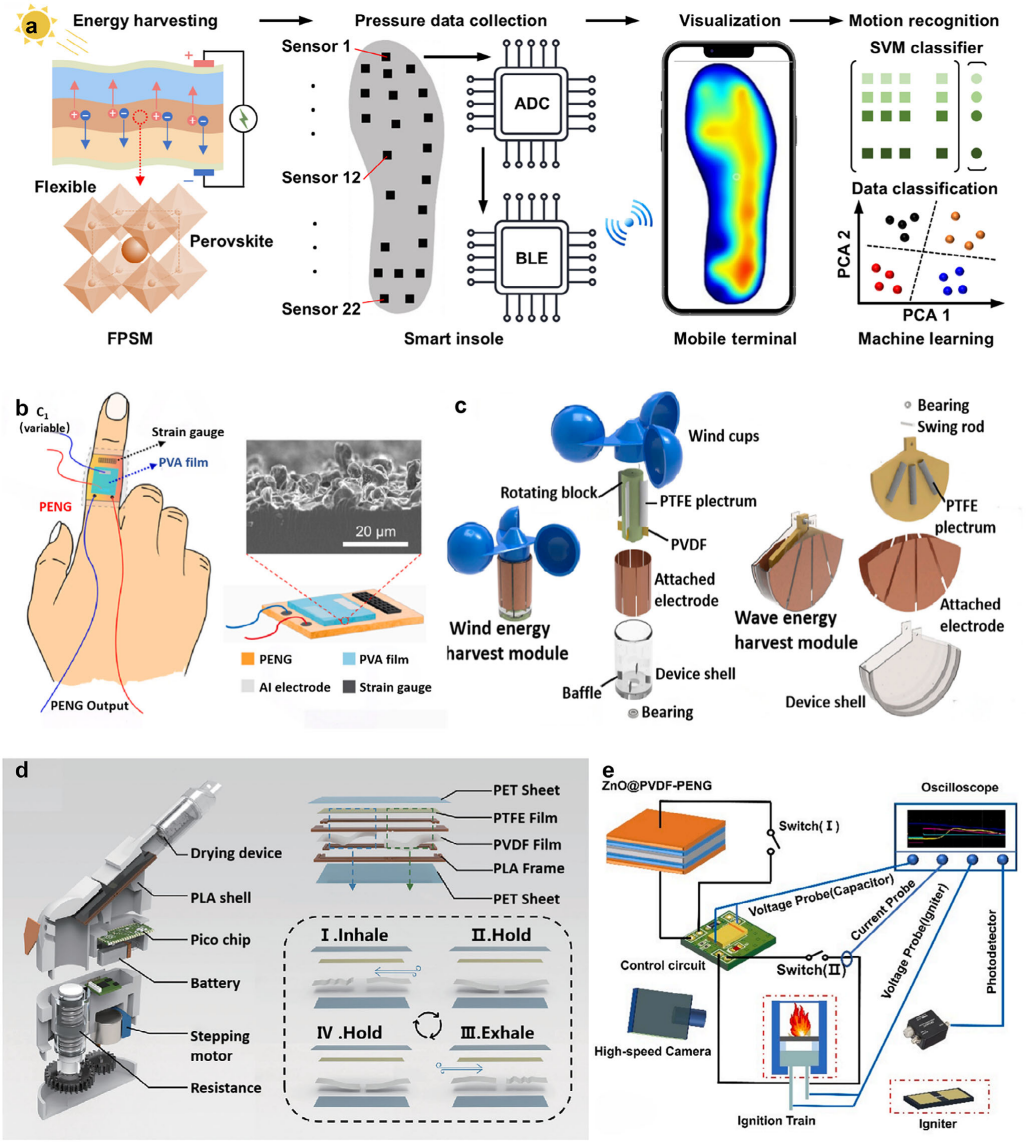
a) Workflow of the smart insole system: A perovskite solar cell harvests energy, foot pressure data are collected via nonlinear synergistic sensing, the pressure distribution is displayed in real time on a mobile terminal, and data classification is ultimately performed using an SVM model. Reproduced with permission.^[^ ^186^ ^]^ Copyright 2025, AAAS. b) Schematic diagram of the PSIW sensor system structure for strain sensing. Reproduced with permission.^[^ ^187^ ^]^ Copyright 2024, Elsevier. c) Schematic diagram of a hybrid wind–wave energy harvesting device based on a triboelectric–piezoelectric composite nanogenerator. Reproduced with permission.^[^ ^188^ ^]^ Copyright 2023, Elsevier. d) Schematic diagram of the sensor module and supporting structure of the intelligent resistive breathing trainer. Reproduced under the terms of the CC‐BY 4.0 license.^[^ ^190^ ^]^ Copyright 2024, The Authors. e) Circuit diagram and schematic illustration of the automatic ignition system. Reproduced with permission.^[^ ^191^ ^]^ Copyright 2025, Elsevier.

Because the native output of PENGs is typically a low‐frequency pulsed AC or a high‐impedance alternating signal, effective utilization requires power management units (PMUs) and energystorage components. The core objectives are ACtoDC conversion, voltage boosting, and impedance matching to the highimpedance piezoelectric source with minimal energy loss.^[^ ^185^ ^]^ For example, Jin et al.^[^ ^186^ ^]^ developed a self‐powered intelligent insole that harvests walkinginduced PENG energy, rectifies and regulates it, and charges a microlithium battery to enable continuous pressure monitoring and humanmotion data acquisition (**Figure** **10**a).^[^ ^186^ ^]^ This system delivers an average power of ≈1.5 mW, supporting continuous gait monitoring and Bluetooth communication. Similarly, Figure 10b illustrates the PENG self‐powered instantaneous wireless (PSIW) sensor system, in which the piezoelectric voltage drives an electronic switch (PEswitch) to optimize impedance matching between the highimpedance PENG and the load. By employing piezoelectricvoltagedriven electronic switching for impedance optimization, the system achieves an energytransfer efficiency of 65.3%, outputs 22 V and 75 µW, and can stably transmit humanmotion information wirelessly over a distance of 30 cm.^[^ ^187^ ^]^

To enhance system reliability under complex environmental conditions, multisource hybridenergy architectures have been developed. As shown in Figure 10c, a TENG–PENG dual‐principle hybrid architecture is proposed, in which windinduced friction, voltagefluctuation, and waveenergy harvesting modules operate synergistically to provide independent or combined outputs, with maximum powers of 3.975 mW, 1.160 mW, and 0.2925 mW, respectively.^[^ ^188^ ^]^ Coupled with an energymanagement circuit, this system can drive LEDs, sensors, and microcalculators, maintaining stable operation over 100 consecutive days, and thereby offering an environmentadaptive powersupply solution for underwater IoT nodes.

At the energystorage level, recent studies have shifted toward hybrid systems combining supercapacitors and solid‐state microbatteries. Ye et al. proposed a microbattery–supercapacitor hybrid storage system (MBSH) integrating PEDOT–TiON nanowires with a porous Ni(OH)_2_ structure.^[^ ^189^ ^]^ The system operates within a 0–1.9 V window, delivering a power density of 77.5 mW cm^−^^2^ and an energy density of 55.6 µWh cm^−^^2^. After 30,000 cycles, the capacity retention remained at 71.8%, enabling reliable operation of timers, LEDs, and thinfilm pressure sensors, and providing high‐power, long lifespan energy storage for miniature electronic devices.

In health monitoring, Xue et al. developed a PENG–TENGbased intelligent respiratorymuscle training system, as shown in Figure 10d.^[^ ^190^ ^]^ Using a Raspberry Pi Pico as the MCU core, the system captures respiratory signals in real time, extracts 12dimensional time and frequencydomain features, and employs PCA to diagnose muscle fatigue with an accuracy of 94.4%. The system subsequently drives a stepper motor to automatically adjust training resistance, forming a personalized, closed‐loop rehabilitation protocol.

Furthermore, Figure 10e illustrates a self‐powered ignition system based on a ZnO@PVDF PENG, highlighting the potential of PENGs in high‐energy transient applications.^[^ ^191^ ^]^ This system can charge a 22 µF capacitor to 15 V within 200 s, drive various ignition devices, and successfully initiate nanoaluminum thermite and explosives, indicating that PENG systems can simultaneously perform low‐power sensing and high‐energy pulsed functions.

In summary, through multisource energy synergy, hybrid energystorage management, intelligent powermanagement circuits, and edgeAI algorithms, PENG systems have evolved from standalone energyharvesting devices into autonomously operating intelligent nodes. This system‐level integration provides a replicable pathway for wearable health monitoring, rehabilitation therapy, intelligent human–machine interaction, and specialized engineering applications.

#### 3.5.3 Intelligent Multi‐Modal Sensing Platform

As sensing demands become increasingly complex, single‑modal sensing is no longer sufficient to meet practical requirements for comprehensive and reliable data acquisition. PENG‑based intelligent multimodal sensing platforms integrate multiple sensing mechanisms, including TENG, photovoltaic, and thermoelectric modules, and combine them with artificial‑intelligence algorithms to achieve data fusion, forming autonomous, self‑powered systems that unify multisignal acquisition and intelligent decision‑making.

At the mechanism‑integration level, combining PENGs with TENGs confers enhanced robustness and power redundancy to the platform. For example, Lu et al. designed a hybrid structure embedding a PENG and a sawtooth TENG, utilizing PTFE microstructures and bent spacers to achieve an open‑circuit voltage of 103 V and an average power of 38 mW.^[^ ^192^ ^]^ Coupled with a long short‑term memory (LSTM) model, the system accurately recognized five distinct gait patterns with an accuracy of 81.8%, demonstrating the stability and predictability of PENG as the primary sensing channel. Similarly, Dong et al. developed a BTO‐PVDF/PDMS sandwich‑structured piezoelectric–triboelectric hybrid nanogenerator (BPP‑HNG), achieving an output voltage of ≈20.5 V and a power density of 130.12 mW m^−^^2^at 20 wt% BTO.^[^ ^193^ ^]^ The device was integrated into a flexible glove, enabling recognition of ten hand gestures and real‑time control of robotic fingers. This system further validates the practicality of hybrid mechanisms in wearable interactive devices.

From a materials‑innovation perspective, researchers have developed composite architectures capable of multiphysics coupling. Li et al. fabricated PVDF‑HFP/Bi_2_Te_3_ core–shell nanofiber membranes (PTCF) via electrospinning combined with magnetron sputtering.^[^ ^194^ ^]^ Under a 5 N load, the membrane generated an open‑circuit piezoelectric voltage of ≈70 V, while a 5 K temperature difference produced a thermoelectric voltage of 2.4 mV. The membrane exhibited good elasticity (Young's modulus ≈8.87 MPa) and high breathability. This piezoelectric–thermoelectric coupled structure enables simultaneous sensing of pressure and temperature, providing a strong foundation for skin‑like sensors and electronic‑skin applications. Meanwhile, Gong et al. introduced fillers such as SiC and FeCl_3_ into PVDF fibers, significantly increasing the β‑phase content.^[^ ^195^ ^]^ As a result, the composite PENG exhibited 12.3‑fold and 10.8‑fold increases in current and voltage, respectively, relative to conventional PVDF PENGs. The high‑performance PENG was integrated into a smart‑sock array, enabling real‑time discrimination of walking, jumping, and distinct gait patterns (e.g., toe‑in and toe‑out), providing a highly sensitive, self‑powered solution for motion monitoring and gait correction.

In the domain of health monitoring, Wang et al. developed a dual‑modal wearable pulse‑detection platform that integrates a PENG and photoplethysmography (PPG).^[^ ^196^ ^]^ The biomimetic fingertip structure ensures stable contact with the skin. The system employs the PENG for continuous prescreening (sensitivity ≈93.41%) and triggers PPG for precise measurement upon detecting anomalies. Combined with a Vision Transformer model, the platform achieves an overall accuracy of 94.95%, with high‑precision stages reaching up to 99.6%. This approach is suitable for long‑term sleep and respiratory monitoring (e.g., sleep apnea), supporting home‑based screening, hospital‑assisted diagnosis, and CPAP therapy evaluation.

AI‐algorithms serve as the primary enabler of advanced cognitive functions in multimodal sensing platforms, playing a pivotal role in multimodal data fusion. In this context, Tan et al. developed an eight‑channel gesture‑recognition system based on a PENG‐TENG hybrid.^[^ ^197^ ^]^ In this platform, the PENG captures high‑force motion signals, whereas the TENG detects light‑touch actions. Signal preprocessing—including Butterworth filtering and independent component analysis (ICA)—and linear discriminant analysis (LDA) classification were implemented on an STM32F4 MCU, achieving a recognition accuracy of 92.6% for 26 sign‑language letters. The platform realizes a closed loop of energy harvesting–multimodal sensing–intelligent recognition, and highlights the central role of PENG in edge‑intelligence applications. Similarly, Yu et al. developed a pressure–temperature dual‑modal sensor (Ag/PMN‐PT/Ag) that combines PENG‑based mechanical‑energy conversion with a pyroelectric response.^[^ ^198^ ^]^ A multilayer perceptron (MLP) neural network was employed to classify six types of thermal and mechanical stimuli, achieving an accuracy of 99.16%. These results demonstrate the substantial potential of co‑designing self‑powered systems, dual‑modal sensing, and AI‑enabled inference for intelligent multimodal platforms.

Although PENG‑based multimodal intelligent sensing systems have achieved substantial progress, several challenges remain for practical deployment. First, the output characteristics of multiple energy‑harvesting mechanisms differ markedly; unstable PENG waveforms and impedance mismatches complicate the design of multi‑port power‑management circuits and reduce overall energy‑integration efficiency. Second, self‑powered edge AI systems must balance recognition accuracy with power consumption, yet existing lightweight models still exhibit inference latency and relatively high energy use on MCUs, limiting long‑term autonomous operation. Third, long‑term reliability remains inadequate, as multimodal devices are prone to interface aging, friction‑layer wear, and susceptibility to temperature and humidity, resulting in output fluctuations and structural fatigue. Systematic encapsulation, interface engineering, and accelerated‑aging evaluation protocols are therefore required.

Overall, PENG‑based multimodal fusion shows substantial potential to evolve into independent intelligent nodes. However, breakthroughs in power management, low‑power AI, structural reliability, and standardization will ultimately determine its feasibility for large‑scale deployment in practice. In summary, integrating PENGs with AI‑enabled signal processing, system‑level energy management, and multimodal sensing platforms provides a viable pathway to flexible, self‑powered intelligent systems. The most promising direction lies in interdisciplinary, integrated design that encompasses coordinated energy harvesting, storage, sensing, and communication modules, alongside AI‑driven, real‑time signal optimization and edge computing, enabling autonomous operation in smart healthcare, the IoT, and infrastructure‑monitoring applications.

## 4 Advanced Energy Harvesting Systems Based on PENG

PENGs are transformative for sustainable energy harvesting, addressing the power constraints of self‐powered electronics such as wearable devices, IoT nodes, and implantable systems. This chapter provides an overview of advanced PENG‐based energy harvesters, ranging from energyconversion fundamentals to integrated self‐charging architectures. The chapter first examines the core electromechanical mechanisms of PENGs and structural designs that enhance efficiency—particularly under low‐frequency excitation, in wearable applications, and during complex deformation. The chapter then explores hybrid harvesters that combine piezoelectric, triboelectric, and electromagnetic mechanisms to broaden available energy sources and functionalities. Finally, the chapter focuses on energystorage integration, highlighting self‐charging power cells (SCPCs) and supercapacitor power cells (SCSPCs) that enable a continuous energy supply via integrated harvesting‐storage architectures. By synthesizing advances in materials, structures, and systems, this chapter offers insights into multifunctional PENG harvesters and their roles in next‐generation self‐powered technologies.

### 4.1 Fundamental Principles and Structural Designs

#### 4.1.1 Principles of Mechanical‐to‐Electrical Energy Conversion

The rapid expansion of wearable and self‐powered devices has created an increasing demand for accessible and sustainable energy storage solutions. The core functionality of self‐powered devices lies in converting environmental or human‐generated inputs into electrical signals for data processing or energy generation.^[^ ^199^, ^200^ ^]^ Conventional wearable technologies predominantly rely on rechargeable batteries, characterized by limited lifespan and constrained sustainability.^[^ ^201^ ^]^ In contrast, PENG‐based self‐supplied energy memory systems can directly convert mechanical energy into electrical energy, potentially offering a promising solution to mitigate the dependency of batteries on external power sources. However, despite the remarkable stability and sustainability of traditional self‐fed energy storage systems, challenges remain in optimizing material properties and extending the operational lifespan of energy storage devices.

#### 4.1.2 Structural Innovations for Enhanced Efficiency

**Figure 11:**
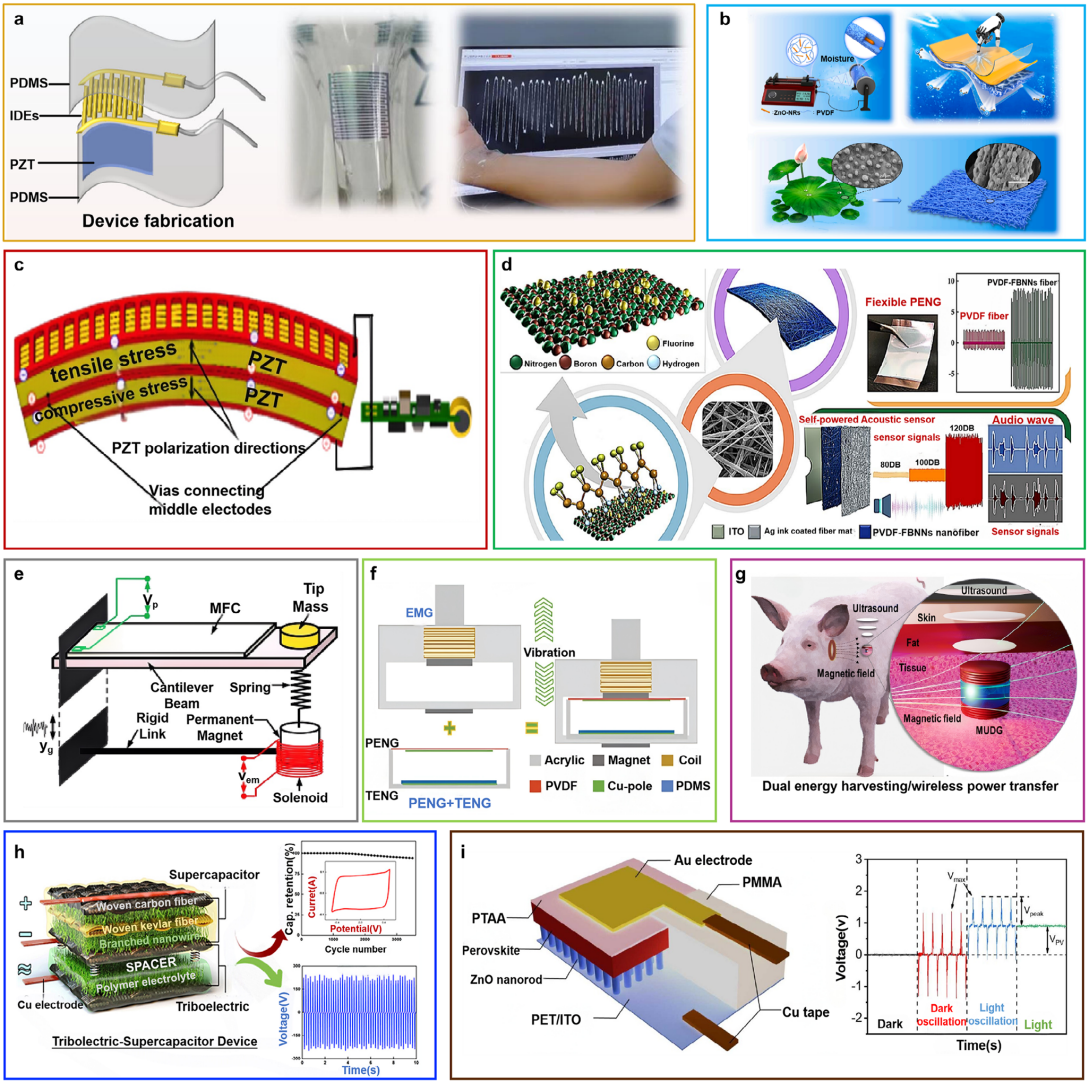
a) The flexible PENG device's design is shown in a comprehensive schematic diagram, which emphasizes the incorporation of interdigital electrodes and the encapsulation in PDMS for increased flexibility and durability. Reproduced with permission.^[^ ^202^ ^]^ Copyright 2025, Springer Nature.b) PAN/TMAB composite fiber felt‐based flexible nanogenerator: self‐powered multifunctional sensor application. Reproduced with permission.^[^ ^203^ ^]^ Copyright 2025, Elsevier. c) Mechanism of operation of a bicrystalline MFC energy harvester. Reproduced with permission.^[^ ^204^ ^]^ Copyright 2022, Elsevier. d) PAN/TMAB composite fiber felt‐based flexible nanogenerator: self‐powered multifunctional sensor application. Reproduced with permission.^[^ ^205^ ^]^ Copyright 2025, Elsevier. e) A schematic illustration. The piezoelectric subsystem is made up of a tip‐loaded unimorph piezo cantilever, whereas the electromagnetic subsystem is made up of a spring‐magnetic mass and solenoid. Reproduced with permission.^[^ ^206^ ^]^ Copyright 2024, Elsevier. f) Harvester assembly step‐by‐step diagram. Reproduced with permission.^[^ ^207^ ^]^ Copyright 2022, Wiley–VCH. g) An illustration of the MUDG gadget for dual energy harvesting (ultrasound and magnetic field) that includes a description of each part.Reproduced with permission.^[^ ^208^ ^]^ Copyright 2024, The Royal Society of Chemistry. h) Triboelectric‐supercapacitor device.Reproduced with permission.^[^ ^53^ ^]^ Copyright 2020, Elsevier. i) Diagrammatic representation of the hybrid energy harvester's structure and signal output in the presence of sunlight. Reproduced under the terms of the CC‐BY 4.0 license.^[^ ^209^ ^]^ Copyright 2025, the authors.

Different structural configurations have a profound impact on the performance and application versatility of PENGs, particularly under low‐frequency excitation, wearable integration, and complex mechanical deformation. Each design contributes unique functional advantages tailored to specific use cases. As illustrated in **Figure** **11**a, an independent (111)‐oriented self‐supporting PbZr_0_._52_Ti_0_._48_O_3_ single‐crystal thin‐film PENG achieves a peak open‐circuit voltage of 12 V. The device maintains stable electrical output when attached to joints such as fingers, wrists, and elbows during bending.^[^ ^202^ ^]^ It exhibits a theoretical sensitivity of 0.58 V Pa^−1^ and a piezoelectric coefficient of ≈585 pm V^−1^. Additionally, its high endurance over 60,000 operational cycles, 3.4% strain tolerance, and a peak power density of 63.5 mW cm^−^^3^ highlight its strong potential for flexible electronics. To achieve moisture resistance and efficient energy harvesting, a microsphere architecture was developed using polyvinylidene fluoride/zinc oxide nanorods (PVDF/ZnO‐NRs) nanofibers (Figure 11b). This structure mimics the microprotrusions of lotus leaves, imparting superhydrophobicity and self‐cleaning behavior. When pressure is applied, the microsphere structure deforms more than a flat film, significantly enhancing electrical conversion efficiency.^[^ ^203^ ^]^ Figure 11c presents an energy harvester powered by animal motion. It consists of a dual‐piezoelectric wafer MFC structure with a self‐powered acoustic sensor embedded between copper electrodes, an epoxy matrix, and polyimide encapsulation. Implanted beneath the skin of a swimming fish, the piezoelectric ceramic fibers (PZT) deform with movement, enabling real‐time energy harvesting, distribution, and closed‐loop power storage.^[^ ^204^ ^]^ In Figure 11d, an electrospun piezoelectric nanogenerator composed of PVDF/BNNS nanofibers converts sound waves into electrical signals by inducing lattice rearrangement within the fiber crystals. Serving as a standalone acoustic sensor, the device responds to multi‐band acoustic frequencies, suggesting valuable applications in environmental noise surveillance and sound recognition.^[^ ^205^ ^]^ A hybrid vibration energy harvester (Figure 11e) combines piezoelectric and electromagnetic mechanisms. Its piezoelectric subsystem comprises a top mass, a piezoelectric layer, and a cantilever beam. When the beam vibrates, bending strains in the microfiber composite material (MFC) generate charges, which are converted into usable energy. Simultaneously, the electromagnetic subsystem—composed of springs, coils, and permanent magnets—induces currents through flux variations at the beam's free end.^[^ ^206^ ^]^

Compared to traditional mechanical designs, magnetic fields offer tunable, noncontact excitation for energy harvesting. Figure 11f illustrates a hybrid harvester integrating electromagnetic (EMG), PENG, and TENG systems to collect energy from human footfalls.^[^ ^207^ ^]^ The device achieves power outputs of 0.168 µW (PENG), 6490 µW (EMG), and 0.09072 mW (TENG) at 4 Hz, sufficient to continuously power 80 LEDs. Figure 11g shows a wireless hybrid harvester that captures energy from magnetic field variations and ultrasonic vibrations. The device employs a pre‐compressed spring and adjustable track to maximize low‐frequency vibration conversion. Its ultrasonic module stimulates piezoelectric elements via mechanical deformation, while a resonant cavity amplifies energy output. Implantation in pig tissue confirms its ability to wirelessly power medical electronics such as pacemakers, harvesting energy from both internal and external biological sources.^[^ ^208^ ^]^

Material selection is critical to achieving robust energy harvesting. As depicted in Figure 11h, a multifunctional system based on woven carbon fiber (WCF) incorporates carbon fiber and PDMS electrodes, with Kevlar serving as a separator.^[^ ^53^ ^]^ It features a friction‐based energy harvesting layer and a supercapacitor for stable current output. Delivering 443.2 V open‐circuit voltage, 0.1325 mA peak current, and 89% efficiency, the system offers exceptional stability. To simultaneously harvest solar and mechanical energy, a hybrid energy harvester (HEH) integrates perovskite layers with ZnO nanoarrays (Figure 11i). The piezoelectric effect in ZnO is triggered by mechanical deformation, while the perovskite layers respond to light exposure. The output increases significantly under concurrent operation. Under an 820 kΩ load, the device generates 320 µW, and energy conversion efficiency improves to 23.7% as impedance decreases from 1 to 0.82 MΩ.^[^ ^209^ ^]^ This innovative electro–optomechanical design points toward new avenues for low‐power, self‐sustaining electronics.

In summary, the principles governing mechanical‐to‐electrical energy conversion and structural optimization are critical determinants of the output performance of PENGs. Studies have demonstrated that selecting appropriate nanomaterials and engineering micro and nanoscale geometries significantly improves energyconversion efficiency and longterm stability. Future development should focus on designing programmable flexible structures and adaptive interfacemodulation strategies to enable efficient energy harvesting in complex operating environments.

### 4.2 Hybrid and Multifunctional Harvesting Systems

#### 4.2.1 Multi‐Mechanism Integrated Harvesters

Electromagnetic induction and the piezoelectric effect—two fundamental energy conversion mechanisms—are widely utilized in energy harvesters due to their applicability across a broad range of vibration scenarios. These mechanisms exhibit strong complementarity, enabling both efficient energy conversion and a reliable continuous power supply.

**Figure 12:**
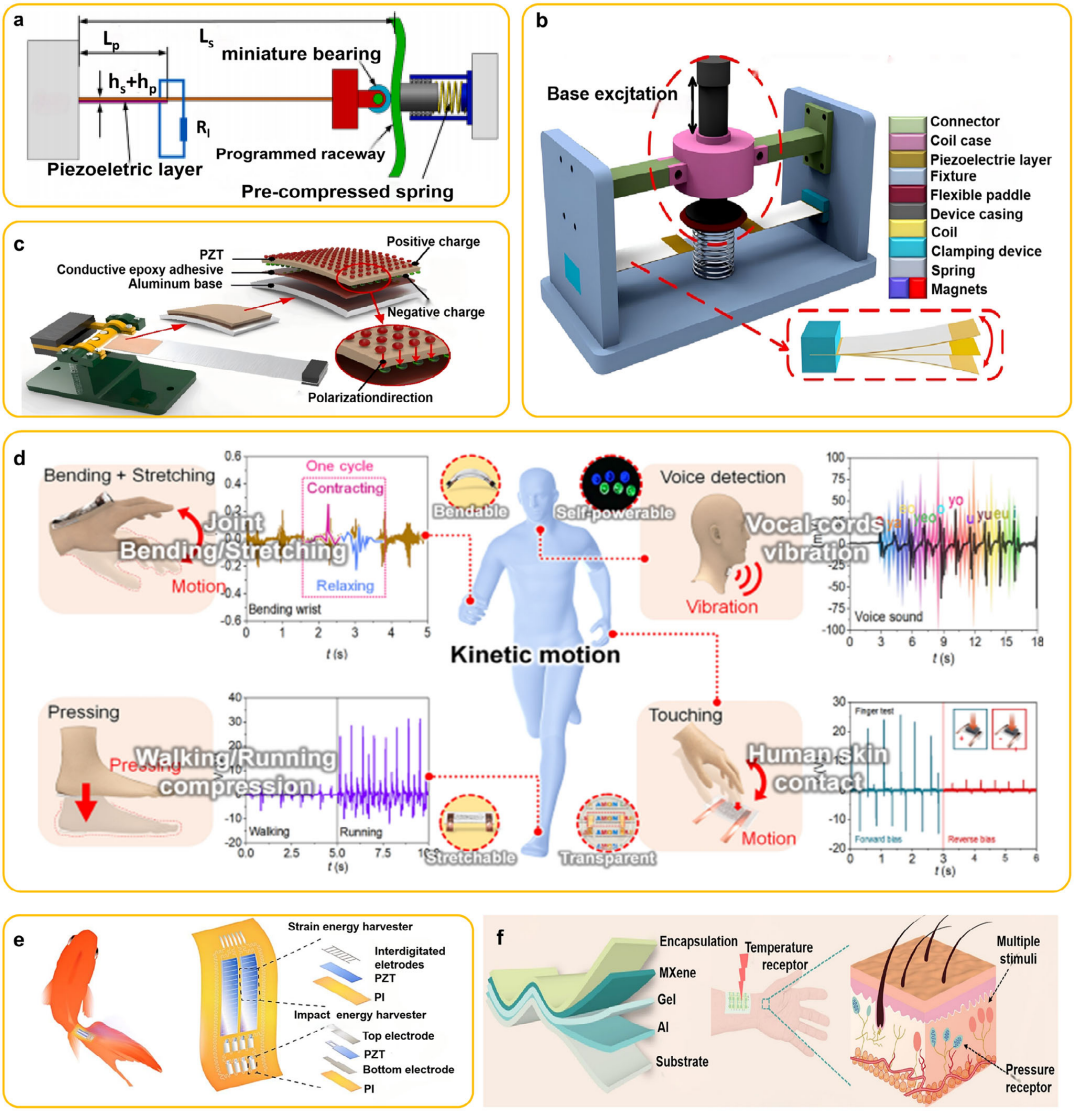
a) An energy harvester's schematic diagram including configurable equilibrium point variants.Reproduced with permission.^[^ ^210^ ^]^ Copyright 2021, Elsevier. b) Diagram of a piezoelectric–electromagnetic energy harvesting device. Reproduced with permission.^[^ ^211^ ^]^ Copyright 2024, Wiley–VCH. c) The bonded piezoelectric sheet's composite structure and operation.Reproduced with permission.^[^ ^212^ ^]^ Copyright 2022, Elsevier. d) All‐nanofiber piezoelectric composite materials' ability to harvest human kinetic energy and their use in motion monitoring. Reproduced with permission.^[^ ^213^ ^]^ Copyright 2024, Springer. e) Schematic structure of the dual modal FWPNG. Reproduced with permission.^[^ ^214^ ^]^ Copyright 2023, American Chemical Society. f) SPDM sensor design concept, schematic diagram of sensing mechanism, and human skin receptor response to temperature and pressure. Reproduced with permission.^[^ ^215^ ^]^ Copyright 2024, Wiley–VCH.

A multi‐stable piezoelectric energy harvester incorporating a programmable equilibrium point configuration is shown in **Figure** **12**a. The structural design consists of a base, pre‐compressed springs, micro‐bearings, a tunable runway, and a piezoelectric layer. The device utilizes its multi‐stable mechanical behavior to convert low‐frequency environmental vibrations into periodic deformation of the piezoelectric element.^[^ ^210^ ^]^ By adjusting the spring pre‐compression force, users can tune the harvester to different vibration intensities and frequencies, thereby optimizing energy capture under variable environmental conditions.

A hybrid energy harvester designed to collect energy from human walking motion is depicted in Figure 12b. This system integrates both piezoelectric and electromagnetic subsystems that operate concurrently to enhance total energy harvesting efficiency. The electromagnetic subsystem includes three cylindrical magnets, coil boxes, induction coils, and sleeves, and functions by detecting magnetic flux variations induced by footstep vibrations.^[^ ^211^ ^]^ Simultaneously, the piezoelectric subsystem comprises flexible piezoelectric beams, PZT ceramic elements, support brackets, and spring‐based reset mechanisms. As the PZT materials deform under mechanical stress, polarization occurs within the crystal lattice, generating surface charges that are harvested as electrical energy. Operating at a vibration frequency of 84 cycles per minute, the device produces outputs of 4.04 V, 4.9 mW (piezoelectric), and 0.57 µW (electromagnetic), which are sufficient to continuously illuminate 60 light‐emitting diodes during human walking.

#### 4.2.2 Smart Multifunctional Platforms

Beyond optimizing the energy conversion efficiency of active materials, refining the internal microstructure and overall system design plays a pivotal role in enhancing the adaptability and multifunctionality of wearable devices. Figure 12c presents a piezoelectric energy harvester tailored for low‐frequency mechanical vibration—a prevalent and continuous energy source in real‐world environments.^[^ ^212^ ^]^ The device consists of a substrate, a conductive epoxy bonding layer, and a piezoelectric PZT layer. Upon mechanical excitation, the harvester exhibits second‐order resonance, realigning its internal dipole moments and generating a potential difference across the material. This mechanism effectively converts mechanical energy into electrical output. The harvester's second‐order intrinsic voltage varies with acceleration and peaks at 22.1 V. At an excitation level of 2.94 m s^−^^2^, it achieves an output voltage of 49.15 V and power of 4.03 mW, demonstrating robust energy harvesting capability.

To simultaneously enable biomechanical energy harvesting and motion monitoring, a piezoelectric nanogenerator was constructed using a BaTiO_3_/P(VDF‐TrFE) composite nanofiber mat, fabricated through electrospinning and electroplating techniques (Figure 12d). The device delivers a high piezoelectric response of 240 V MPa^−1^, surpassing performance benchmarks of comparable materials. Featuring a breathable non‐woven architecture and MF‐based electrodes, it balances user comfort, low visual detectability, 80% optical transparency, and mechanical flexibility. Finger tapping induces voltage spikes up to 25.7 V, attributed to the synergistic interplay of triboelectric and piezoelectric effects. This device facilitates real‐time tracking of physiological signals such as joint movement, foot strikes, vocal cord vibrations, and skin deformation, while concurrently harvesting biomechanical energy. These capabilities advance the development of multifunctional, energy‐autonomous wearable electronics.^[^ ^213^ ^]^

Driven by the increasing demand for integrated energy harvesting and environmental sensing in next‐generation wearables, multifunctional harvesters are now engineered to simultaneously perform solar conversion, mechanical energy capture, and parameter detection. A dual‐mode, fish‐shaped wearable piezoelectric nanogenerator (FWPNG) is introduced in Figure 12e to fulfill these demands.^[^ ^214^ ^]^ The strain‐based module consists of a polyimide (PI) substrate, PZT, and interdigitated electrodes (IDEs) that generate current through structural deformation caused by swimming motion. The impact‐based module comprises PZT and PI layers sandwiched between top and bottom electrodes, converting hydrodynamic impacts into transient electrical pulses. This dual‐mode configuration enables efficient mechanical energy harvesting across a wide frequency spectrum and contributes to the realization of self‐powered underwater bio‐wearables for precision aquatic motion sensing.

Figure 12f illustrates a self‐powered dual‐temperature–pressure (SPDM) sensor based on a MXene‐enhanced composite hydrogel.^[^ ^215^ ^]^ This architecture integrates a compressible ionic gel electrolyte and utilizes potential differences between MXene and aluminum electrodes to generate signals. External pressure deforms the contact interface, eliciting a piezoelectric response, while the temperature sensitivity exploits the negative temperature coefficient of MXene, whereby increased ion mobility at higher temperatures alters the electrical response. By applying machine learning algorithms, the system effectively differentiates between pressure and thermal stimuli with 99.1% classification accuracy. The sensor operates reliably across a wide temperature range (5–75 °C) and pressure span (0–800 kPa), while offering excellent mechanical flexibility and skin compatibility—making it highly suitable for intelligent physiological monitoring in diverse environmental conditions.

This section examines multimechanismcoupled energy harvesting and systemlevel integration. Multifunctional platforms that integrate piezoelectric, triboelectric, and additional transduction mechanisms can deliver a stable power supply across diverse environmental conditions. The development of intelligent harvesting platforms equipped with environmental recognition and autonomous modeswitching capabilities represents a key advance toward achieving a continuous energy supply for wearable devices and distributed sensor networks.

### 4.3 Integrated Energy Conversion and Storage Systems

#### 4.3.1 Self‐Charging Power Cells (SCPCs)

The rapid development of IoT technologies and wearable electronics has introduced new demands for energy supply systems, particularly the need for continuous power delivery and minimal maintenance.^[^ ^216^ ^]^ Harvesting energy from ambient sources—such as wind, solar radiation, mechanical vibrations, and human motion—offers a promising approach to fulfilling these demands.^[^ ^217^ ^]^ Traditionally, energy conversion and storage have been implemented in separate devices, which increases system complexity and causes energy losses at the interfaces.^[^ ^218^ ^]^ Integrating these functions within a single unit can simultaneously stabilize and store the intermittent energy harvested from the environment, while enhancing overall energy utilization efficiency by reducing losses associated with interconnects and energy management circuitry.^[^ ^219^ ^]^

**Figure 13:**
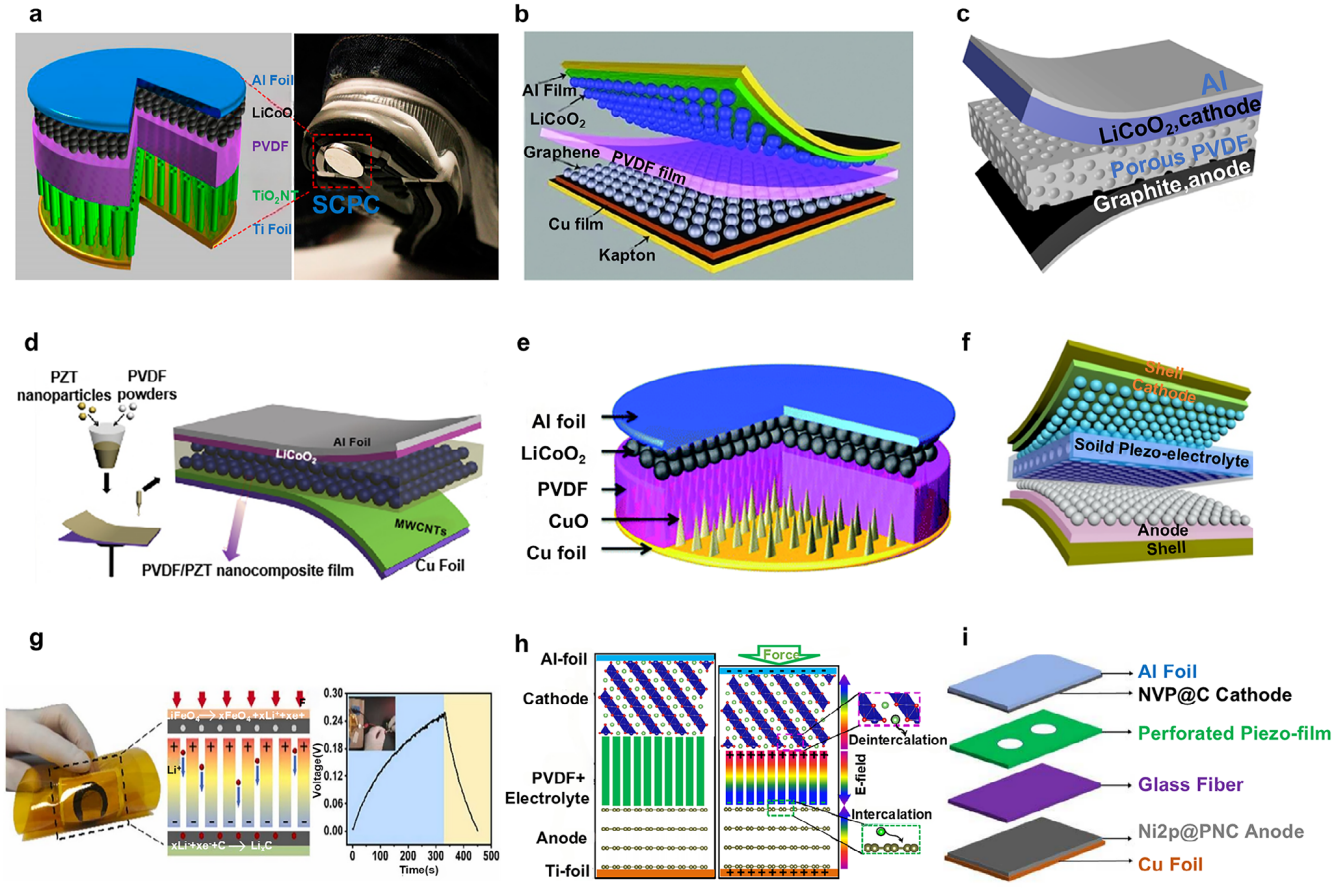
SCPCs based on PENGs. a) The first SCPC, which sealed in a stainless‐steel 2016‐coin‐type cell. Reproduced with permission.^[^ ^15^ ^]^ Copyright 2012, American Chemical Society. b) The first flexible SCPC which using Kapton boards as the shells. Reproduced with permission.^[^ ^220^ ^]^ Copyright 2013, Wiley–VCH. c) Highly porous piezoelectric PVDF membranes based SCPC. Reproduced with permission.^[^ ^222^ ^]^ Copyright 2015, Elsevier. d) PVDF–PZT nanocomposite film‐based SCPC. Reproduced with permission.^[^ ^223^ ^]^ Copyright 2014, IOP Publishing. e) CuO/PVDF nanocomposite anode‐based SCPC. Reproduced with permission.^[^ ^224^ ^]^ Copyright 2013, Royal Society of Chemistry. f) Piezo‐electrolyte‐based all‐solid‐state flexible SCPC. Reproduced with permission.^[^ ^20^ ^]^ Copyright 2017, Elsevier. g) The electrospinning P(VDF‐TrFE) porous membranes based SCPC. Reproduced with permission.^[^ ^225^ ^]^ Copyright 2021, Elsevier. h) Modelling and simulation of piezoelectrically driven SCPC. Reproduced with permission.^[^ ^226^ ^]^ Copyright 2017, American Chemical Society. i) The self‐charging sodium‐ion battery. Reproduced with permission.^[^ ^22^ ^]^ Copyright 2019, Elsevier.

Xue et al. pioneered the development of an SCPC by integrating piezoelectric materials with electrochemical energy‐storage devices.^[^ ^15^ ^]^ In this design, a polarized PVDF film—exhibiting both ion permeability and piezoelectricity—replaces the conventional polyethylene (PE) separator in a lithium‐ion battery (LIB), as illustrated in **Figure** **13**a. Under mechanical actuation, the piezoelectric field generated by the PVDF drives lithium‐ion (Li⁺) migration from the cathode to the anode, thereby converting mechanical energy directly into stored electrochemical energy. When subjected to a mechanical force of 45 N at 2.3 Hz, the SCPC's voltage increased from 327 mV to 395 mV within 240 s, demonstrating significantly higher efficiency than conventional systems that employ separate harvesting and storage components. **Table** **4** summarizes the structural configurations and output characteristics of PENG‐based SCPCs.

**Table 4:** Comparison of component materials and output properties of SCPCs based on PENG.

Positive electrode materials	Piezoelectric materials	Negative electrode material	Self‐charging performance	Energy storage capacity	Reference
Al foil‐Li_2_CoO_2_	PVDF film	Ti foil‐TiO_2_ NT	327 to 395 mV in 240 s (applied force is 45 N at 2.3 Hz)	0.036 µAh	[15]
	PVDF film	Cu foil‐Graphene	500 to 832 mV in 500 s (applied force is 34 N at 1.0 Hz)	0.266 µAh	[220]
	Mesoporous PVDF film (ZnO anoparticles as sacrificing pore‐forming agents)	Cu foil‐Graphene	1.2 to1.4 V in 200 s (applied energy is 282mJ at 1Hz)	0.4 µAh	[222]
	Mesoporous PVDF film (ZnO NWs as sacrificing pore‐forming agents)	Cu foil‐Graphene	160 to 299 mV in 250 s (applied force is 34N at 1.8Hz)	0.173 µAh.	[221]
	PVDF–PZT nanocomposite film	Cu foil‐ MCNTs	210 to 297.6 mV in 240 s (applied force is 10 N at 1.5 Hz)	0.01 µAh	[223]
	CuO/PVDF nanocomposite anode	Cu foil‐ CuO	50 to 144 mV in 240 s (applied force is 18N at 1 Hz)	0.0247 µAh	[224]
	The solid piezo‐electrolyte	Cu foil‐Graphene	25 to 473 mV in 240 s (applied force is 30 N at 1.0 Hz)	0.118 µAh	[20]
	PVDF/BCT‐BZT nanocomposite film	Cu foil‐CNTs	800 to 1100 mV in 600 s (applied energy is 294 mJ at 0.5 Hz)	1.4 µAh	[229]
Al foil‐LiFeO_4_	P(VDF‐TrFE)/PEG/TPU nanofiber membrane	Cu foil‐Graphene	70 to 240 mV in 330 s (applied force is 6 N at 1.0 Hz)	0.092 µAh	[225]
Al foil‐ NVP@C	KNN@SEBS piezo‐film	Cu foil‐Ni_2_P@PNC	0.19 to 0.65 V in 150 s (applied force is 10 N at 2.0 Hz)	None	[22]
Sodium foil	BaTiO_3_‐NTs‐P (VDF‐HFP)‐NaClO_4_	Al foil‐ Na_0.71_Co_0.96_O_2_	2.0 to 3.21 V in 100 h under 5N static pressure	1.132 mAh	[230]

Building on this concept, subsequent studies have focused on improving SCPC performance through structural optimization and material innovations. To reduce energy losses arising from rigid electrodes and casings, Xue et al. introduced a flexible SCPC that employed graphene‐based electrodes and Kapton enclosures in place of conventional stainless‐steel frameworks, as schematically illustrated in Figure  13b.^[^ ^220^ ^]^ The resulting device maintained stable capacity and electrolyte integrity during repeated mechanical deformation, confirming its robustness for wearable applications. Enhancing ion transport represents another effective strategy for performance improvement. Porous PVDF separators have been reported to accelerate ion mobility.^[^ ^221^, ^222^ ^]^ Kim et al. fabricated porous piezoelectric membranes via a ZnO‐particle template‐assisted method (Figure  13c).^[^ ^222^ ^]^ SEM images revealed interconnected mesoporous domains that provided abundant ion‐transport channels, thereby improving charge–discharge performance compared with low‐porosity membranes. Zhang et al. further enhanced SCPC performance by incorporating a PVDF–PZT nanocomposite film as a piezo‐separator.^[^ ^223^ ^]^ By dispersing PZT nanoparticles within a PVDF matrix and fabricating the composite film via spin‐coating, they achieved a higher piezoelectric potential due to strain confinement and enhanced porosity for ion conduction (Figure  13d). The resulting SCPC exhibited a storage capacity 2.5 times greater than that of devices using pure PVDF films. Xue et al. subsequently fabricated an integrated SCPC utilizing a CuO/PVDF nanoarray structure serving as the piezo‐anode.^[^ ^224^ ^]^ PVDF gel was spin‐coated onto chemically etched CuO nanoarrays, creating a high‐interfacial‐area interface that enhanced the efficiency of piezoelectric field utilization, as shown in Figure  13e. Under a 1 Hz compression force of 18 N for 240 s, the device achieved a storage capacity of 0.0247 mAh and an energy output of 6.12 mJ—approximately threefold higher than that of its non‐integrated counterpart.

Despite the widespread use of liquid electrolytes, challenges such as leakage, poor electromechanical stability, and chemical volatility limit the reliability of SCPCs. He et al. addressed these limitations by developing the first all‐solid‐state SCPC (Figure  13f), incorporating a piezo‐separator that simultaneously served as a solid electrolyte.^[^ ^20^ ^]^ This component was prepared by incorporating LiPF_6_ solution into a mesoporous PVDF matrix synthesized via a SiO_2_‐microsphere templating method. The sealed SCPC charged from 105 to 220 mV within 300 s under a 30 N force at 1 Hz and maintained consistent performance over 20 bending cycles, demonstrating both mechanical durability and self‐charging capability.

Yu et al. recently demonstrated a flexible SCPC employing a porous P(VDF‐TrFE) nanofiber film fabricated via electrospinning with PEG additives to induce hierarchical porosity (Figure  13g).^[^ ^225^ ^]^ This multifunctional film acted as both the piezoelectric separator and mechanical support for the electrodes, onto which electrodes were knife‐coated on both sides. Sealed within a flexible enclosure, the integrated device effectively charged from 70 to 240 mV within 330 s under periodic finger tapping (6 N at 1 Hz), outperforming several conventional self‐powered systems.

To elucidate the role of the piezoelectric field in electrochemical processes, Wang et al. employed density functional theory (DFT) simulations to model a representative SCPC structure.^[^ ^226^ ^]^ As illustrated in Figure  13h, to maintain chemical equilibrium between electrode materials and the electrolyte when subjected to an external mechanical force, the piezopotential of the separator drives Li⁺ deintercalation (LiCoO_2_ → Li_1_₋_x_CoO_2_ + xLi⁺ + xe^−^) at the cathode and intercalation (C_6_ + xLi⁺ + xe^−^ → Li_x_C_6_) at the anode. The results reveal that the piezoelectric potential reduces the energy barriers for lithium‐ion deintercalation and intercalation, thereby facilitating the self‐charging mechanism.

In contrast to LIB‐based SCPCs, Zhou et al. developed an SIB‐based SCPC that incorporated a perforated piezo‐separator into a flexible SIB architecture (Figure  13i).^[^ ^22^ ^]^ SIBs offer advantages including high energy density, long cycle life, cost‐effectiveness, and environmental compatibility.^[^ ^227^, ^228^ ^]^ The high ionic conductivity and low solvation energy of Na⁺ ions make them particularly well‐suited for wearable power supply applications. The SIB‐based SCPC exhibited excellent self‐charging performance, reaching ≈0.65 V through mechanical bending within 150 s or palm tapping within 300 s, highlighting its potential as an effective hybrid device for simultaneously harvesting and storing energy.

#### 4.3.2 Self‐Charging Supercapacitor Power Cells (SCSPCs)

In recent years, supercapacitors (SCs) have emerged as viable alternatives to batteries in specific applications due to their superior power density.^[^ ^231^ ^]^ SCs are generally categorized into two types: electrochemical double‐layer capacitors (EDLCs) and pseudocapacitors. They offer significantly higher power density than batteries, as their charge storage relies on surface reactions at the electrode–electrolyte interface rather than on ion diffusion within the bulk material.^[^ ^232^ ^]^ Compared with batteries, SCs offer advantages including rapid charge–discharge rates, exceptional long‐term cyclability, nontoxic or minimally toxic electrolytes, and enhanced safety.^[^ ^233^, ^234^ ^]^ These characteristics position SCs as promising next‐generation energy‐storage devices and implantable power‐supply systems.^[^ ^54^, ^235^ ^]^

**Figure 14:**
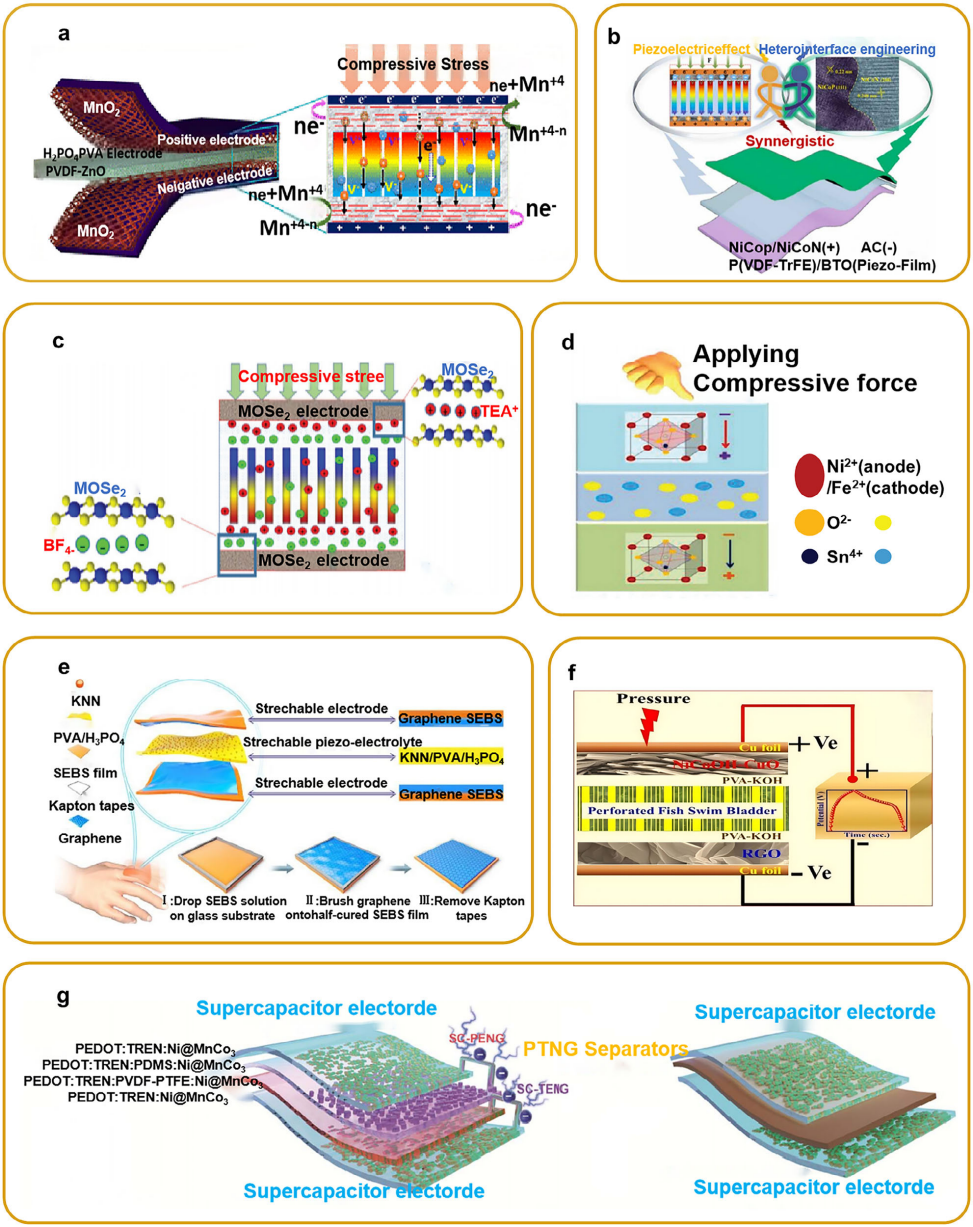
SCSPCs based on PENGs. a) The first SCSPC, which uses MnO_2_ nanowires as positive and negative electrodes and PVDF‐ZnO film as a separator. Reproduced with permission.^[^ ^54^ ^]^ Copyright 2015, American Chemical Society. b) The SCSPC, which uses nanoarchitecture with heterointerface enhanced performance. Reproduced with permission.^[^ ^238^ ^]^ Copyright 2021, Elsevier. c) The solid‐state SCSPC, which comprising 2D MoSe_2_ as an energy storing electrode with PVDF‐co‐HFP/TEABF_4_ ion gelled PVDF/NaNbO_3_ as the piezopolymer electrolyte. Reproduced with permission.^[^ ^244^ ^]^ Copyright 2018, Wiley–VCH. d) The SCSPC, which uses lead‐free perovskites as piezoelectrodes and PVA‐KOH film as ionogelled electrolyte. Reproduced with permission.^[^ ^241^ ^]^ Copyright 2022, Wiley–VCH. e) Highly stretchable SCSPC which using graphenecoated elastic styrene–ethylene–butylene–styrene electrodes and KNN/PVA/H_3_PO_4_‐based piezoelectric electrolyte. Reproduced with permission.^[^ ^242^ ^]^ Copyright 2017, Royal Society of Chemistry. f) The SCSPC, which using fish swim bladder as an efficient natural bio‐piezoelectric separator. Reproduced with permission.^[^ ^97^ ^]^ Copyright 2017, Elsevier. g) A piezo–triboelectric hybrid effect‐driven SCSPC which can operate at low temperatures. Reproduced with permission.^[^ ^243^ ^]^ Copyright 2023, Royal Society of Chemistry.

Inspired by prior studies on SCPCs^[^ ^15^ ^]^ and self‐charging electrochemical biocapacitors,^[^ ^236^ ^]^ Ramadoss et al. were the first to fabricate a SCSPC based on a PENG.^[^ ^54^ ^]^ Their design employed PVDF–ZnO as the piezoelectric separator, PVA–H_3_PO_4_ as the gel electrolyte, and electrochemically active MnO_2_ nanowires as electrodes, as illustrated in **Figure** **14**a. This innovation successfully integrated energy‐harvesting and storage functionalities within a single supercapacitor device. The SCSPC was capable of charging to 110 mV within 300 s under palm impact and demonstrated its utility by powering a green LED. A typical SCSPC comprises three main components: electrodes, separators, and electrolytes. The performance of SCSPCs is primarily determined by the choice of electrode materials, piezoelectric materials, and device architecture. Electrode materials define electrochemical characteristics, piezoelectric materials influence power‐generation efficiency, and the device structure governs integration, thereby influencing the device's responsiveness to external mechanical stimuli.

Heterointerface engineering can significantly improve intrinsic reaction kinetics and structural stability through electronic coupling effects and interfacial synergistic interactions.^[^ ^237^ ^]^ Gao et al. developed a solid‐state SCSPC comprising a NiCoP/NiCoN cathode, an activated carbon (AC) anode, a PVA/KOH gel electrolyte, and a P(VDF‐TrFE)/BTO piezo‐film separator, as depicted in Figure  14b.^[^ ^238^ ^]^ The NiCoP/NiCoN heterostructure, synthesized via a one‐step in situ phosphonitridation process, exhibited a high areal capacitance of 3544 mF cm^−^^2^ at 1 mA cm^−^^2^ and excellent cycling stability, retaining 84.38% of its initial capacitance after 5000 cycles at 20 mA cm^−^^2^. This improved performance is attributed to the abundant heterointerfaces and lattice defects, which expose additional interfacial electroactive sites and facilitate faster electrolyte‐ion transport.^[^ ^239^ ^]^ Furthermore, the NiCoP/NiCoN‐based SCSPC delivered outstanding energy‐storage performance (62.1 Wh kg^−1^ at 850 W kg^−1^) and superior self‐charging capability, achieving a voltage increase of 133 mV within 146 s under a compressive force of 35 N.

Nanostructured electrode materials, by virtue of their nanoscale dimensions and large specific surface areas, provide abundant electroactive sites for electrochemical reactions, thereby enhancing their suitability for energy‐storage applications. **Table** **5** presents a comparative analysis of the constituent materials and output performance of SCSCs, a key class of PENG‐based devices. Pazhamalai et al. demonstrated the fabrication of an SCSPC employing 2D MoSe_2_ nanosheet electrodes, which combined double‐layer capacitance and pseudocapacitance to enhance energy‐storage capability (Figure  14c). The device incorporated an electrospun PVDF/NaNbO_3_ nanofibrous mat as the piezo‐separator, which offered increased ionic‐conduction pathways due to its porosity, and a PVDF‐co‐HFP‐based ionogel as the electrolyte, which exhibited higher charge‐transfer resistance than ionic‐liquid electrolytes. Benefiting from these material configurations, the SCSPC achieved a specific capacitance of 18.93 mF cm^−^^2^, a specific energy of 37.90 mJ cm^−^^2^, and a power density of 268.91 µW cm^−^^2^ at a constant discharge current of 0.5 mA. The device could be charged to 708 mV under a compressive force of 30 N within 100 s, demonstrating its superior performance.

**Table 5:** Comparison of component materials and output properties of part SCSCs based on PENG.

Electrode	Separator	Electrolyte	Capacitance	Self‐charging performance	Energy density	Power density	Cyclic stability	Reference
MnO_2_‐rGO	PVDF‐ZnO‐rGO	PVA‐H_3_PO_4_	7.6 F g^−1^	stored 1.5 × 10^−3^ mC charge in 100 s (under a hand‐tapping compressional force)	10.34 mWh kg^−1^	193.6 µW cm^−2^	81 % after 1000 cycles	[245]
BNNT–CNF/ZnO	PVA‐KOH solid gel	300 F g^−1^ at 1 A g^−1^	/	37.5 W h kg^−1^.	0.9 kW kg^−1^	/	97% after 5000 cycles	[246]
Graphene	PVDF‐ TEABF_4_	28.46 F g^−1^ at 31.63 mF cm^−2^	charged 112 mV in 250 s (applied force 20 N)	35.58 Wh kg^−1^	7500 W kg^−1^	/	91% after 5000 cycles	[247]
WS_2_@ PPy	PVDF film	H_2_SO_4_/PVA	337.7 F g^−1^ at 1 A g^−1^	charged 880 mV in 60 s (under finger pressing)	26.38 Wh kg^−1^	1874 W kg^−1^	/	[248]
Carbon fabric	PAN	PVA/ H_2_SO_4_	15.5 mF cm^−2^ at 0.1 mA cm^−2^	charged 17.8 mV in 300s (applied force 30N at 1.5Hz)	37.60 mJ cm^−2^	1.01 mW cm^−2^	91.7 % after 5000 compressive cycles	[249]
NiCoOH‐CuO@Cu foil	Fish swim	PVA–KOH	165.1 F g^−1^ at 1 A g^−1^	charged 150 mV in 80 s (under human finger imparting at 1.65 Hz)	51.6 Wh kg^−1^	750 W kg^−1^	94.5 % after 8000 cycles	[97]
NiSnO_3_ as anode,FeSnO_3_ as cathode	Common separator	PVA‐KOH	144 F g^−1^ at 1.67 A g^−1^	charged 200 mV (under the thumb pressing)	45 W h kg^−1^	1.25 kW kg^−1^	90 % after 10000 cycles	[241]
CoFe_2_O@ activated carbon cloth	Filter paper	PVA‐KCl‐BaTiO_3_	2.86 mF cm^−2^ at 0.090 mA cm^−2^	charged 120 mV in 420s (bent 180° at 1 Hz)	/	/	95 % after 11,000 cycle	[250]
MnO_2_	PVDF‐ZnO	PVA/H_3_PO_4_	455 mF g^−1^ at 0.04 mA cm^−2^	charged 110 mV in 300 s (under palm impact)	91 mWh kg^−1^	3.9 kW kg^−1^	/	[54]
MoS_2_	Nafion	1.81 mF cm^−2^ at 10 mV s^−1^ scan rate	charged 243 mV in 600 s (applied mechanical force)	542 µJ cm^−2^	0.1 mW cm^−2^	/	95% after 10000 cycles	[251]
PPy/Ni/RS	Rochelle salt	6 m KOH	1262.5 F g^−1^ at 1.5 A g^−1^	charged 700 mV in 15s (under 30 N compressive force)	166.23 Wh kg ^−1^	0.24 kW kg^−1^	99 % after 10000 cycles	[252]
Ni as anode,Mg‐Co as cathode	PVA/ZnO/KOH	70 F g^−1^ at 1.5 A g^−1^	charged 55 mV in 150 s (applied force 5 N)	5.46 Wh kg^−1^	0.56 KW kg^−1^	/	96.5 % after 10000 cycles	[240]
PDMS‐rGO/C	P(VDF‐TrFE)	PVA/H_2_SO_4_	44.6 µF cm^−2^ at 250 µA cm^−2^	charged 450 mV (bent to 90°)	0.078 µW h cm^−2^	0.025 mW cm^−2^	98 % after 20 000 cycles	[253]
Graphene	PTA‐PVDF	184.94 mF cm^−2^ at 1.0 mA constant discharge current	charged 110 mV in 200 s (applied force 2 N)	59.18 mJ cm^−2^	0.177 mW cm^−2^	/	86.4 % after 5000 cycles	[254]
MXene	MXene–PVDF	H_2_SO_4_‐PVA	61 mF cm^−2^ at 5 mV s^−1^ scan rate	/	24.9 mJ cm^−2^	1.3 mW cm^−2^	89 % after 4500 cycles	[255]
CuCoNiO_4_	CuCoNiO_4_/PVDF	PVA/ H_3_PO_4_	15.5 mF cm^−2^ at 0.1 mA cm^−2^	charged 810 mV in 220 s (bent 180° at 1 Hz)	1.9 mW h cm^−2^	0.12 mW cm^−2^	98 % after 10000 cycles	[24]

Conventional symmetric supercapacitors (SCs), consisting of electrodes made from identical materials, are constrained by a narrow potential window, which limits the overall energy density of the device. In contrast, asymmetric supercapacitors utilize different electrode materials to efficiently exploit the potential difference between electrodes, thereby increasing the cell voltage.^[^ ^232^ ^]^ Verma et al. developed an asymmetric SCSPC using Ni and Mg–Co nanowire‐based binder‐free electrodes synthesized through electrodeposition.^[^ ^240^ ^]^ The Ni anode exhibited a specific capacitance of 114.28 F g^−1^ at 2 A g^−1^ with 100% retention after 10 000 cycles, whereas the Mg–Co cathode achieved a specific capacitance of 317.5 F g^−1^ with 96.5% retention. The fabricated device demonstrated excellent performance and reproducibility, achieving a voltage of 99 mV under thumb‐press‐induced compressive force.

SCSPCs typically harvest external mechanical energy using piezoelectric separators or piezoelectric electrolytes. Padha et al. introduced an all‐solid‐state SCSPC comprising piezoelectric perovskite‐based electrodes (NiSnO_3_ and FeSnO_3_ prepared via a sol–gel method) and a PVA–KOH gel electrolyte.^[^ ^241^ ^]^ These environmentally friendly and biocompatible electrodes are well‐suited for wearable electronics (Figure  14d). The resulting piezoelectric potential drives ion migration within the electrolyte, initiating electrochemical redox reactions. The device self‐charges to a maximum of 266 mV under bending, twisting, and compressive forces.

Selecting appropriate materials to ensure reliability, versatility, and compatibility remains a major development focus for SCSPCs. With the rapid advancement of wearable electronics, the demand for flexibility and stretchability in energy‐storage devices has grown considerably. Zhou et al. fabricated a stretchable SCSPC by sandwiching a KNN/PVA/H_3_PO_4_‐based piezo‐electrolyte film between stretchable graphene‐coated elastic styrene–ethylene–butylene–styrene (SEBS) electrodes.^[^ ^242^ ^]^ Figure  14e shows the structure and preparation steps of the stretchable graphene/SEBS electrode. The device exhibited 300% stretchability, enabling effective accommodation of mechanical strain and maintaining efficient self‐charging capability. It charged to 1.0 V within 300 s of palm patting at 2 Hz, and to 0.8 V within 40 s of repeated stretching at 1 Hz, indicating potential for powering stretchable electronic devices.

Maitra et al. developed a natural bio‐piezoelectric‐driven SCSPC employing nickel–cobalt double hydroxide nanopiles with copper oxide nanoflakes as electrodes, and a perforated fish swim bladder impregnated with a PVA–KOH gel electrolyte as the separator (Figure  14f).^[^ ^97^ ^]^ This environmentally friendly and fluorine‐free separator enabled the device to charge up to 281.3 mV under human finger input at 1.65 Hz within 80 s. Eight serially connected SCSPCs successfully powered various portable electronic devices and illuminated four red LEDs. Selvam et al. introduced a piezo–triboelectric nanogenerator (PTNG)‐based SCSPC capable of operating at extremely low temperatures (−80 °C). Utilizing ionic‐liquid‐based electrolytes, the device achieved a high specific capacitance of 317 F g^−1^ at 1 A g^−1^ and maintained 65% capacitance retention at −80 °C.^[^ ^243^ ^]^ The PTNG separator enabled simultaneous piezoelectric and triboelectric energy conversion, as illustrated in Figure  14g.

**Figure 15:**
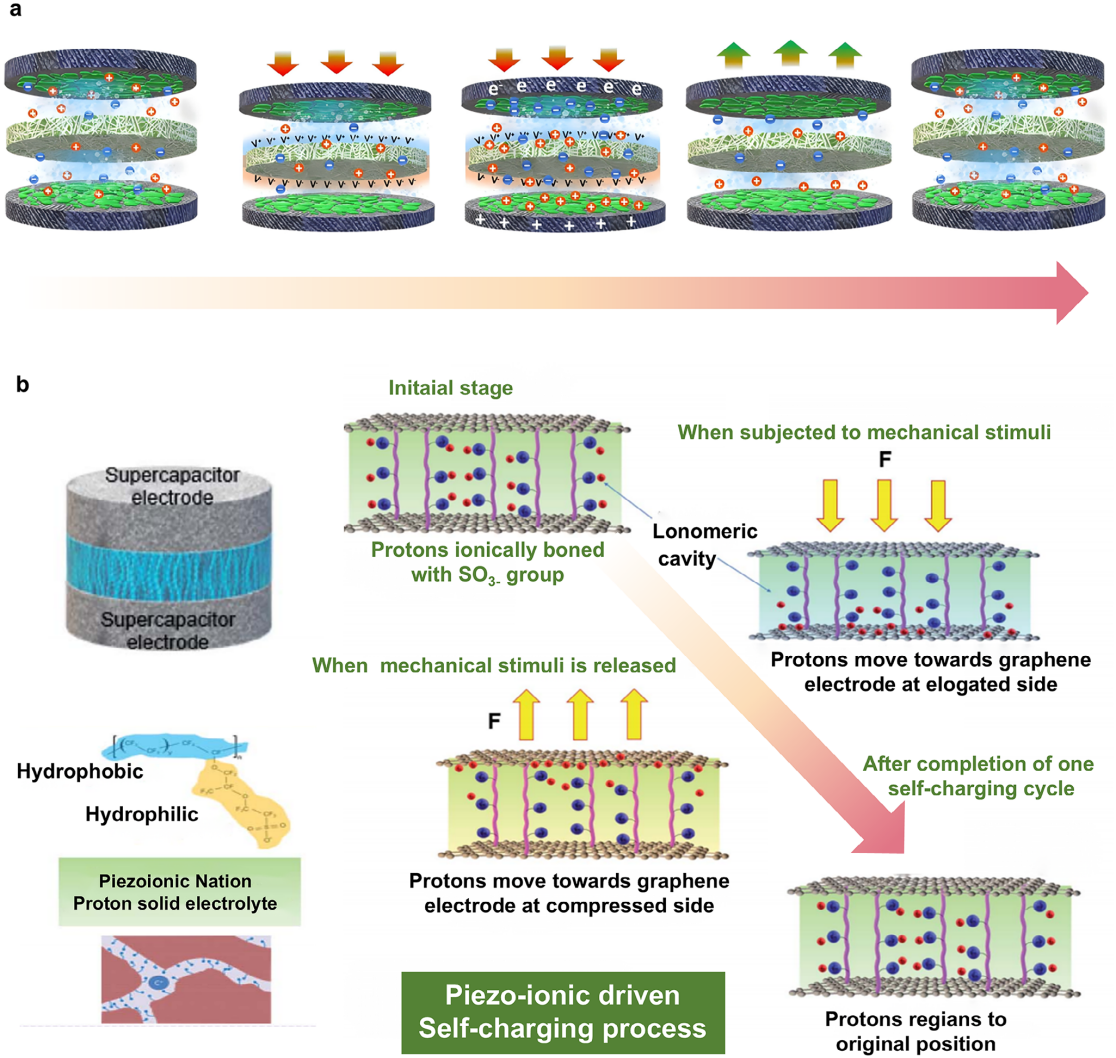
a) The mechanism of the self‐charging process is discussed via “piezoelectrochemical effect” with the aid of piezoelectrochemical spectroscopy measurements. Reproduced with permission.^[^ ^256^ ^]^ Copyright 2020, Springer. b) The mechanism of peozo‐ionic driven self‐charging process. Reproduced with permission.^[^ ^257^ ^]^ Copyright 2022, Royal Society of Chemistry.

To date, the piezoelectrochemical processes governing SCSPC operation remain insufficiently understood.^[^ ^244^ ^]^ Krishnamoorthy et al.^[^ ^256^ ^]^ employed piezoelectrochemical spectroscopy (PECS) to directly probe the “piezoelectrochemical effect” in siloxene‐based SCSPCs, revealing a charge‐state‐dependent response under mechanical deformation. **Figure** **15**a presents the cyclic voltammetry results, confirming enhanced charge injection from the electrolyte to the electrode surfaces under applied mechanical force. Subsequently, Krishnamoorthy et al.^[^ ^257^ ^]^ further advanced SCSPC design by incorporating Nafion polyelectrolyte films serving as dual‐functional separators. Figure 15b depicts the piezo‐ionic‐driven self‐charging process in SCSPCs, highlighting the underlying proton‐migration mechanisms activated under mechanical stimulation. These findings offer valuable insights for decoupling mechano‐ and electrochemical gating mechanisms to enhance SCSPC performance.

#### 4.3.3 Impedance Matching Challenges and Power Management Strategies

Although integrating PENGs with SCPCs or SCSPCs offers a promising route to continuous energy supply, the fundamental bottleneck is the severe impedance mismatch between the high‑voltage, low‑current pulses generated by PENGs and the low‑voltage, high‑current characteristics required by supercapacitors. Under mechanical excitation, PENGs typically produce transient high voltages ranging from tens to over one hundred volts, while delivering extremely low currents. In contrast, supercapacitors operate optimally at low voltages with relatively high and stable charge–discharge currents. This disparity leads to a significant reduction in energy‑conversion efficiency when the two components are directly coupled.

To address this issue, the power‑management circuit (PMC) plays a pivotal role in integrated systems. By incorporating rectification, impedance‑matching, and voltage‑regulation modules, the PMC converts the high‑voltage pulses generated by PENGs into a direct‑current (DC) voltage suitable for charging supercapacitors, while switch‑control strategies and multisource energy routing enable efficient energy integration.^[^ ^258^ ^]^ Yang et al. proposed an AC‐DC rectifier topology with switched rectification, which efficiently converts the high‑voltage AC pulses from PENGs into DC signals suitable for supercapacitor charging.^[^ ^259^ ^]^ Impedance‑matching techniques such as SSHI (synchronous switch harvesting on inductor) and SECE (synchronous electric charge extraction) offer significant advantages in this context. For example, Lo et al. employed an SP‑SECE circuit in a magnetically triggered rotational PENG, achieving efficient impedance matching through metal‐oxide‐semiconductor (MOS) switching and electromechanical damping.^[^ ^260^ ^]^ The circuit achieved an efficiency of 81%, with its self‑consumption accounting for only 2.8% of the harvested power, thereby providing an effective solution for self‑powered sensing in rotating machinery.

From a materials–device co‑design perspective, Das et al. developed a flexible SCPC–PENG system based on La_2_CuMnO_6_‐PVDF composite fibers, in which the PENG delivered an output voltage of 21 V and a current of 1.5 µA.^[^ ^261^ ^]^ The SCPC exhibited a specific capacitance of 111 Fg^−1^ at a scan rate of 5 mV s^−1^ and maintained 95% capacity retention after 2000 charge–discharge cycles. With 30 s of thumb pressing, the system self‑charged to 750 mV, demonstrating efficient, integrated energy harvesting and storage for wearable electronics. Qin et al. proposed a passive PMC for pulsed TENGs, consisting solely of a rectifier bridge, an inductor, and a capacitor, with no active components.^[^ ^262^ ^]^ This design achieved an equivalent input impedance approaching zero, with a simulated energy‑storage efficiency of 83.6% and an experimental efficiency of 57.8%. It successfully powered a microsensor and a calculator, offering a simple, low‑cost solution suited to pulse‑based energy‑harvesting scenarios.

In summary, the PMC is an indispensable core component for the deep integration between PENGs and supercapacitors. Future research should focus on dynamic PMC architectures, multisource input management, and AI‑enabled energy‑scheduling strategies to overcome efficiency and stability limitations in integrated PENG–SC systems, thereby enabling long‑term, reliable operation in wearable electronics, the IoT, and implantable devices. Integrating PENGs with SCPCs and SCSPCs can significantly enhance energy‑storage and utilization efficiencies, enabling continuous, low‑maintenance, self‑powered systems. Future development should emphasize highly integrated, intelligent strategies, including coordinated optimization of energy harvesting, storage, and release; rapid charge–discharge capabilities; and high‑power‑density designs, to provide sustainable energy for flexible, self‑powered devices and distributed sensor nodes.

## 5 Summary and Outlook

This review first synthesizes the latest research advances on PENGs, with particular emphasis on their application potential in flexible electronics and self‑powered sensing. The review further provides a critical analysis of key technical challenges in PENG technologies and proposes actionable solutions and interdisciplinary research pathways to facilitate translation to scalable applications and autonomous systems. PENGs represent an advanced energy harvesting technology with significant potential for powering low‐energy systems, energy storage, and integration into multifunctional platforms such as high‐capacity energy storage units and flexible sensors. By utilizing the piezoelectric effect, PENGs convert mechanical energy from environmental sources—such as vibrations, pressure, and human motion—into electrical energy, offering an eco‐friendly and highly efficient energy solution for self‐sustaining electronic devices. With the continuous evolution of nanotechnology and system integration techniques, PENGs have undergone substantial progress in material engineering and structural optimization, laying the groundwork for applications in areas such as environmental monitoring, wearable electronics, and the IoT.

Advances in material selection have led to marked improvements in PENG performance. Organic polymers (e.g., PVDF‑based) are favored in flexible electronics for their processability and mechanical compliance, whereas inorganic materials (e.g., ZnO and BaTiO_3_) are valued for their superior piezoelectric properties. The use of flexible substrates has further expanded the applicability of PENGs across diverse operating environments. Meanwhile, micro‑ and nanostructuring techniques, together with advanced fabrication methods such as 3D printing and laser‑induced structuring, have enhanced the suitability of PENGs for capturing complex environmental and human mechanical signals. Deep integration with SCSPCs and SCPCs significantly increases system energy autonomy, providing maintenance‑free power for IoT nodes and wearable devices.

Despite continuous performance improvements, PENG technologies still face critical bottlenecks in translation to practical applications. First, output power and energy density remain low, making it challenging to directly power self‑powered sensors, microscale energy‑storage devices, or complex electronic systems. Addressing this challenge requires complementary strategies: i) developing materials with enhanced piezoelectric properties, optimizing micro‑ and nanostructures, and implementing mechanical‑amplification designs; and ii) integrating efficient power‑management circuits and energy‑scheduling strategies to collectively improve energy harvesting and output capabilities.

Second, long‑term stability and environmental adaptability remain inadequate. Devices are prone to performance degradation under cyclic loading or in high‑temperature/high‑humidity environments. Mitigation strategies include integrating self‑healing functionalities into materials, strengthening interfacial protection, employing waterproof and corrosion‑resistant encapsulation at the system level, and conducting extensive durability testing and in‑situ validations to ensure reliability.

Third, device‑to‑device consistency and scalability remain challenging. Laboratory‑fabricated devices often exhibit performance variability and high costs during scale‑up and mass production. Advancing scalable manufacturing—such as printed electronics, 3D printing, and laser processing—together with standardized materials and modular designs can improve product uniformity, reduce costs, and support large‑scale deployment.

Finally, system‑level power management and energy‑storage integration remain limited, constraining the practical use of PENGs in self‑powered systems. The typical output—intermittent, high‑impedance, and low‑amplitude signals—cannot directly supply power. Developing efficient power‑management circuits coupled with energy‑storage units is necessary to establish an integrated “harvest‐store‐supply” module, augmented by intelligent algorithms for real‑time energy allocation and optimization.

Furthermore, applications of PENGs in multimodal sensing and intelligent systems remain at an exploratory stage. Although promising results have been reported in pressure, gas, and physiological‑signal detection, multisignal fusion and the construction of fully functional intelligent platforms have yet to be realized. Future efforts should focus on deep integration of PENGs with sensing, AI analytics, and wireless‑communication modules to develop intelligent systems that unify data acquisition, power supply, and communication. Standardization and demonstration projects should be promoted for scenarios including health monitoring, infrastructure, and environmental surveillance.

Looking forward, the development of PENGs can focus on several key directions: designing piezoelectric materials informed by materials science and AI; optimizing micro‑ and nanoscale fabrication and structures; developing wearable and implantable medical devices; investigating intelligent energy‑management algorithms; and planning system‑level integration and commercialization pathways. Through interdisciplinary convergence, a closed‑loop design—from material selection and structural optimization to system integration and practical deployment—can be achieved, accelerating the large‑scale application of PENGs in healthcare, smart cities, and infrastructure monitoring. In the short term, emphasis can be placed on low‑power sensing and wearable health monitoring; in the medium term, expansion toward self‑powered medical monitoring systems is anticipated; and in the long term, deployment of large‑scale arrays for infrastructure and industrial applications is envisioned, progressively establishing an industrial chain from core devices and functional modules to complete systems.

## Conflict of Interest

The authors declare no conflict of interest.
